# NKG2D-mediated detection of metabolically stressed hepatocytes by innate-like T cells is essential for initiation of NASH and fibrosis

**DOI:** 10.1126/sciimmunol.add1599

**Published:** 2023-09-29

**Authors:** Sonja Marinović, Maja Lenartić, Karlo Mladenić, Marko Šestan, Inga Kavazović, Ante Benić, Mia Krapić, Lukas Rindlisbacher, Maja Cokarić Brdovčak, Colin Sparano, Gioana Litscher, Tamara Turk Wensveen, Ivana Mikolašević, Dora Fučkar Čupić, Lidija Bilić-Zulle, Aleksander Steinle, Ari Waisman, Adrian Hayday, Sonia Tugues, Burkhard Becher, Bojan Polić, Felix M. Wensveen

**Affiliations:** 1 Department of Histology and Embryology, Faculty of Medicine University of Rijeka, Croatia; 2 Institute of Experimental Immunology, University of Zürich, Zürich, Switzerland; 3 Center for proteomics, Faculty of Medicine University of Rijeka, Croatia; 4 Department of Internal Medicine, Faculty of Medicine University of Rijeka, Croatia; 5 Center for Diabetes, Endocrinology and Cardiometabolism, Thallassotherapia, Opatija; 6 Dept. of General Pathology and Pathological Anatomy, Faculty of Medicine Univ. of Rijeka, Croatia; 7 Clinical Department of Laboratory Diagnosis, Clinical Hospital Center Rijeka, Rijeka, Croatia; 8 Institute for Molecular Medicine, Goethe-University, Frankfurt am Main, Germany; 9 Institute for Molecular Biology, University Medical Center, Mainz, Germany; 10 Department of Immunobiology, King’s College London, UK

**Keywords:** NKG2D, NKG2D ligands, MAFLD, NAFLD, IL-17A, NASH, metabolic cellular stress, immunosensing, inflammation, fibrosis, liver, hepatocytes, γδ T cells, innate-like lymphocytes

## Abstract

Metabolic-associated fatty liver disease (MAFLD) is a spectrum of clinical manifestations ranging from benign steatosis to cirrhosis. A key event in the pathophysiology of MAFLD is the development of non-alcoholic steatohepatitis (NASH), which can potentially lead to fibrosis and hepatocellular carcinoma, but the triggers of MAFLD-associated inflammation are not well understood. We have observed that lipid accumulation in hepatocytes induces expression of ligands specific to the activating immune receptor NKG2D. Tissue-resident innate-like T cells, most notably γδ T cells, are activated through NKG2D and secrete IL-17A. IL-17A licenses hepatocytes to produce chemokines that recruit proinflammatory cells into the liver, which causes NASH and fibrosis. NKG2D-deficient mice did not develop fibrosis in dietary models of NASH and had a decreased incidence of hepatic tumors. Importantly, the frequency of IL-17A^+^ γδ T cells in the blood of MAFLD patients correlated directly with liver pathology. Our findings identify a key molecular mechanism through which stressed hepatocytes trigger inflammation in context of MAFLD.

## Introduction

Metabolic-associated fatty liver disease (MAFLD), previously known as non-alcoholic fatty liver disease (NAFLD), is considered the hepatic manifestation of metabolic syndrome and affects approximately one quarter of the global adult population (1–3). MAFLD is a spectrum of clinical entities ranging from simple steatosis to cirrhosis. Whereas many patients only suffer from benign steatosis (metabolic-associated fatty liver; MAFL), some progress to non-alcoholic steatohepatitis (NASH). NASH is characterized by a dramatic increase in inflammatory cells in the liver, which drives the development of fibrosis and can ultimately cause cirrhosis and liver failure. Due to limited treatment options, MAFLD/NASH is quickly becoming one of the leading causes of liver-associated deaths worldwide. A better understanding of the molecular triggers that drive the transition of steatosis to hepatitis and cirrhosis is therefore imperative (4), but the early metabolic stress-specific signals that initiate inflammation in the liver remain largely unknown.

The pathophysiology of NASH is thought to require multiple triggers, including fat accumulation, gut microbiome disbalance, and oxidative stress (2), but general consensus is that the key event in the transition of MAFL to NASH is the initiation of inflammation (2). The events leading up to inflammation are therefore of great clinical interest. CD4^+^ T_H_17 cells and CD8^+^ T cells have been shown to mediate NASH-associated liver fibrosis in both mice and humans (5–9). However, these cells are recruited in response to an established inflammatory milieu and therefore do not initiate immune cell accumulation in the liver. Instead, tissue resident immune cells appear more likely to sense early metabolic stress and induce the inflammatory response (10). Innate like T cells, such as mucosa associated invariant T cells (MAIT) and γδ T cells, have been shown to be involved in the pathophysiology of NASH. In humans and mice, NASH is associated with type 3 inflammation, characterized by the by the production of the cytokine IL-17A, most notably by mucosal-associated invariant T (MAIT) cells and γδ T cells (11–13). In response to T cell receptor (TCR) engagement, these cells were shown to secrete IL-17A and promote liver fibrosis (14). However, TCR triggering alone is typically insufficient for the full activation of these cells (15), and engagement of other activating receptors through stress ligands usually plays a prominent role in immunosurveillance mediated by tissue-resident innate-like lymphocytes (16). The role of stress ligands in the development of NASH is currently unclear.

Several mechanisms have been associated with immune cell activation in response to metabolic stress. We have previously shown that hypertrophic adipocytes upregulate ligands of the activating NK cell receptor NCR1 in obesity (17). As a response, tissue-resident NK cells proliferated and secreted IFN-γ which triggered macrophage polarization and local inflammation (17). In the liver, hepatic stress caused by hepatitis B and C virus infection was associated with the induction of ligands for the activating receptor NKG2D (18, 19). NKG2D is constitutively expressed on many immune cells, most notably innate and innate-like lymphocytes such as NK, MAIT, and γδ T cells, but its ligands are typically only induced in tissues in response to stress (20). Upon NKG2D engagement, immune cells mediate cytotoxic responses and/or produce cytokines, and this receptor has been shown to play a role in many pathologies (20, 21). In a mouse model for hepatotoxicity mediated by injection of carbon tetrachloride, NKG2D ligands (NKG2D-L) were shown to be induced and could drive NK cell-mediated killing of hepatic stellate cells (22). However, whether NKG2D plays a functional role in NASH is currently unknown.

Here, we aimed to elucidate which molecular signal triggers MAFL-to-NASH transition in fatty liver disease. We observed that both induction of NKG2D ligands and IL-17A expression correlate with the early stages of human NASH. Using a dietary model for NASH developed in our lab, we show that metabolic stress in hepatocytes promotes NKG2D-L expression, which is detected by tissue-resident innate-like T cells, most notably γδ T cells. Their secretion of IL-17A early during disease pathogenesis in response to NKG2D engagement licenses hepatocytes to produce chemokines and attract pro-inflammatory myeloid cells. Deficiency of NKG2D or IL-17A receptor expression on hepatocytes resulted in a considerable reduction in liver inflammation and fibrosis. Importantly, IL-17A expression by innate-like T cells in the blood of patients with MAFLD correlated positively with liver pathology. Our findings identify a key molecular mechanism that mediates the transition of MAFL to NASH with potential applications as a diagnostic biomarker and therapeutic target.

## Results

### MAFLD is associated with an increase of NKG2D ligands and IL-17A-expressing cells in the human liver

**Figure 1:**
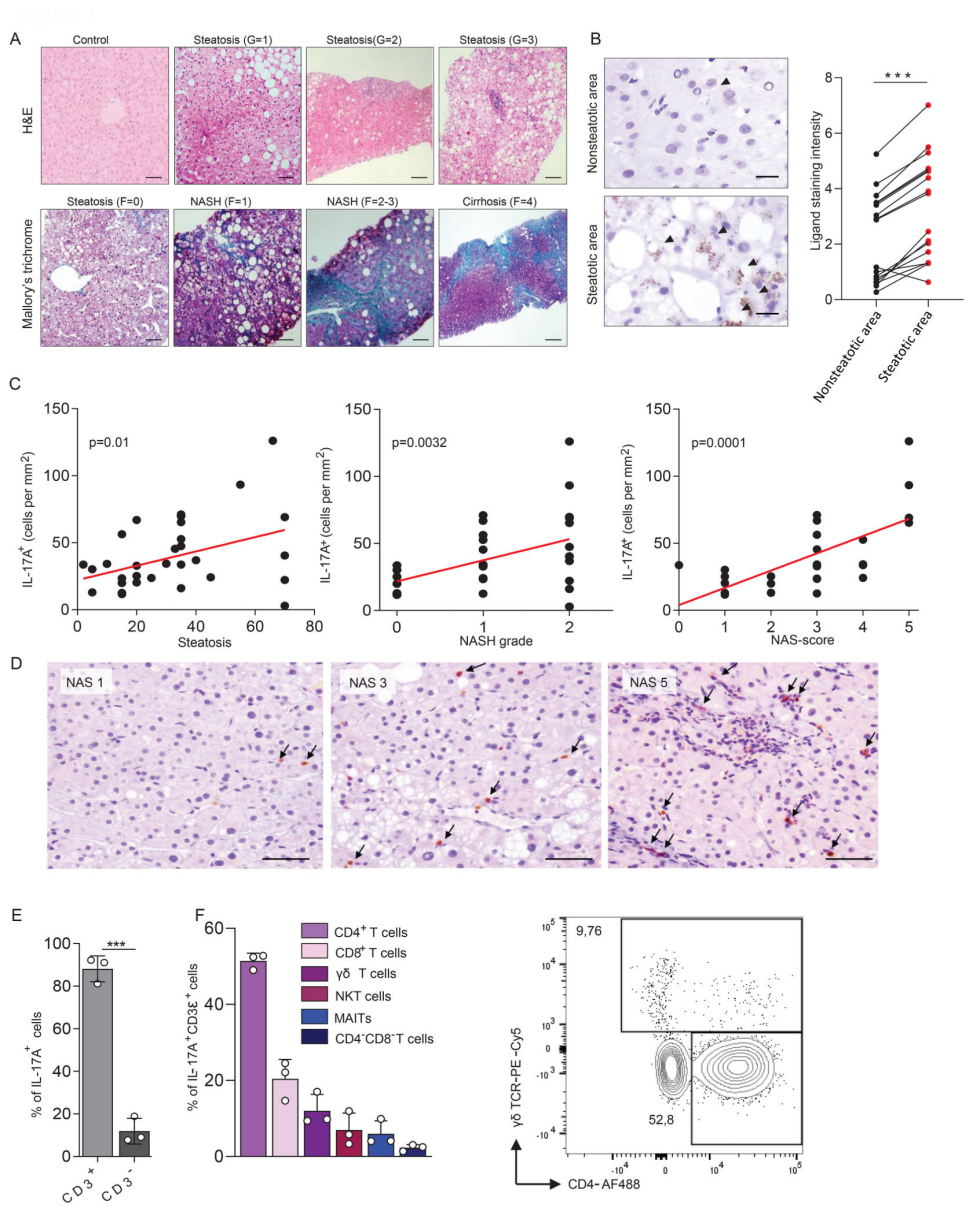
MALFD is associated with an increase of NKG2D ligands and IL-17A expressing cells in the liver (a) Representative histological slides of various stages of liver disease in human liver biopsies. (top) slides were stained with hematoxylin and eosin (H&E) and show a sample from a healthy individual and from MAFLD patients with mild (G=1), moderate (G=2), or severe (G=3) steatosis with ballooning and inflammation. (bottom) slides were stained with Mallory`s trichrome and show liver tissue without fibrosis (F=0) or with perisinusoidal and pericellular fibrosis without (F=1) or together with extensive portal fibrosis (F=2-3) or with cirrhosis (F=4). Scale bars indicate 50 µm for control, steatosis (G=1), steatosis (F=0), NASH (F=1); 100 µm for steatosis (G=2), steatosis (G=3), NASH (F=2-3); 250 µm for cirrhosis (F=4). (b) Human biopsy material of patients with MAFLD was stained for the NKG2D ligands MICA/B. Shown is NKG2D-L expression in steatotic and non-steatotic regions within the same patient (n=17). Scale bars indicate 20 µm. Arrowheads indicate a positive signal. (c-d) Human biopsy material of patients with MAFLD was stained for IL-17A. (c) The number of IL-17A-producing cells was correlated with the level of steatosis, grade of NASH, or the NAS-score (n=35). (d) Representative staining for NAS scores 1, 3 and 5. Scale bars indicate 50µm. (e-f) Quantification of IL-17A-producing cells in biopsies of human livers by flow cytometry. (e) Quantification of CD3^+^ and CD3^-^ cells within the IL-17^+^ population (n=3). (f) Quantification of IL-17A-producing T cells within the CD3^+^IL-17A^+^ cell pool (n=3). Representative plot shows cells gated for IL-17A^+^CD3^+^TCRαV7.2^-^CD56^-^. Statistical significance was determined by Wilcoxon signed-rank test (b), linear regression (c), or unpaired t-test (e). Statistical significance was defined as *p<0.05; **p<0.01; ***p<0.001.

Human MAFLD is associated with steatosis, inflammation, and fibrosis. We analyzed histological sections of liver biopsies from patients that were diagnosed with MAFLD to characterize disease progression. We observed that both steatosis and fibrosis often start in zone I of the liver lobules around the hepatic artery, where oxygenation is highest, and most oxygen radicals are expected to be generated. From here, pathology spreads outwards towards zone III (Fig. 1a). Late-stage steatosis and fibrosis were usually accompanied by diffuse foci of inflammatory cells, which are indicative of NASH.

MAFLD generates both metabolic and inflammatory stress in hepatocytes. We therefore investigated whether ligands of the activating stress receptor NKG2D are induced in liver tissue from patients with MAFLD. Immunohistochemical staining revealed that the NKG2D ligands MICA/B are upregulated specifically in sections most affected by steatosis compared to healthy sections of the same tissue (Fig. 1b and Fig. S1a). We did not observe induction of the NKG2D ligands ULBP1 or ULBP3 in livers of patients. MAFLD has been associated with type 3 inflammation, so we investigated if IL-17A expression is induced in liver biopsy materials of patients with MAFLD and whether this correlates with the disease severity score (23). We observed that the frequency of IL-17A expressing cells positively correlated with both the level of steatosis and liver inflammation (Fig. 1c). Notably, we observed that the increase in IL-17A expressing cells highly correlated with the early stages of MAFLD (NAS score 3-5), whereas it decreased again in later stages of the disease (NAS scores 6 and 7) (Fig. 1c,d and Fig. S1b).

To determine which cells produce IL-17A in human livers, we first made use of publicly available single cell RNA sequencing data of human liver biopsy material (24). This showed that *Rorc*, the transcription factor mediating IL-17A expression, is predominantly expressed by T cells (Fig. S1c). In addition, we found that *Rorc* was expressed in a subpopulation that also expressed *Klrk1*, the gene encoding NKG2D. To confirm that Rorc expression also translated into IL-17A production, immune cells were isolated from fresh biopsy material and stimulated in vitro with PMA and Ionomycin. Cytokine producing cells were identified by flow cytometry. We observed that IL-17A was almost exclusively produced by CD3^+^ cells (Fig. 1e). Of these, most cells had a CD4^+^ Th17 profile. In addition, we observed that CD8 T cells and γδ T cells were prominent sources of IL-17A (Fig. 1f and Fig. S1d).

In summary, we find that human MAFLD is associated with an upregulation of NKG2D ligands and an increase in the frequency of IL-17A producing cells, especially in the early stages of the disease. This suggests that a causal relationship may exist between these two parameters.

### Lipid accumulation in murine hepatocytes induces upregulation of NKG2D ligands

**Figure 2:**
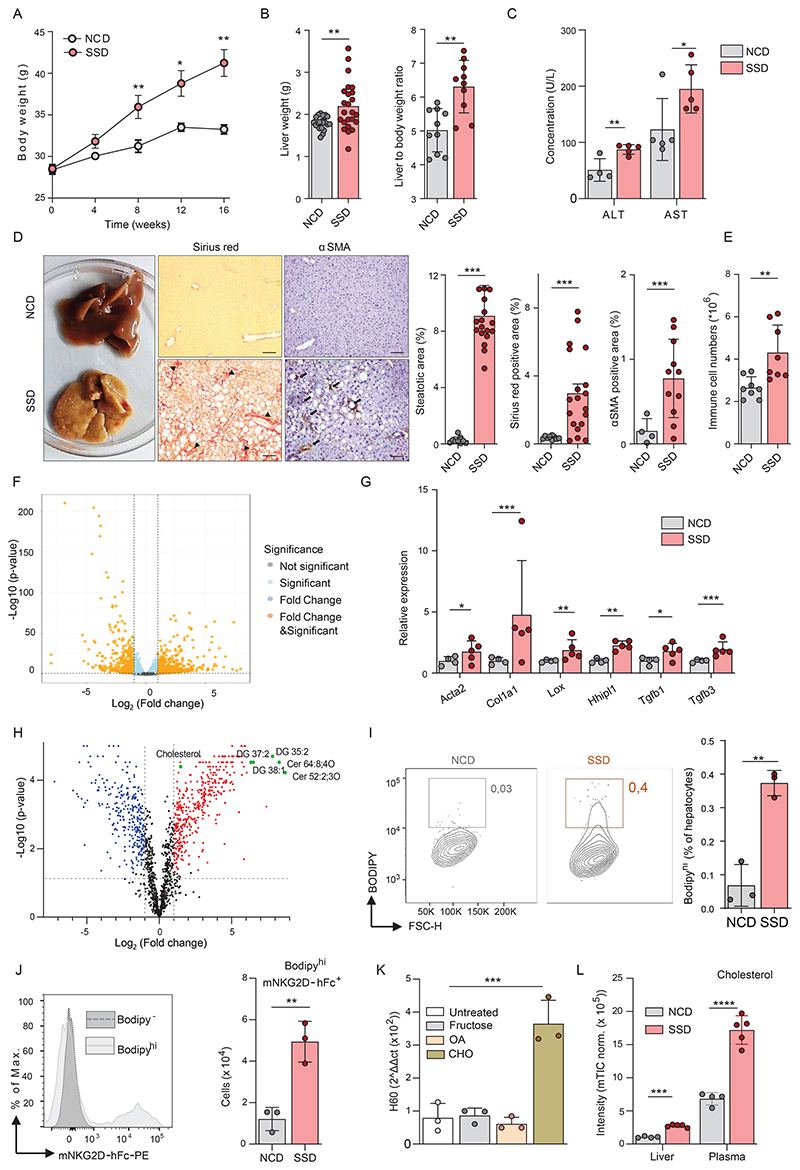
Lipid accumulation in murine hepatocytes induces upregulation of NKG2D ligands. (a-e) WT mice were fed a Steatosis and Steatohepatitis Diet (SSD) or a Normal Chow Diet (NCD) for 2 to 16 weeks. (a) Total body weight over time (n=20). (b) Liver weight and the liver to body weight ratio at 16 weeks (n=22-24). (c) Serum AST and ALT levels after 16 weeks (n=5). (d) Representative liver slides (200x) of fibrosis and hepatic stellate cell activation (αSMA) after 16 weeks of SSD feeding. Macroscopic changes in livers of SSD diet-fed mice showing yellow/gray firm parenchyma indicative for liver steatosis and fibrosis. Arrowheads indicate collagen accumulation. Arrows indicate αSMA^+^ cells. Bar diagrams show the quantification of histology slides (n=4-19). Scale bars indicate 20 µm. (e) Quantification of immune cells (CD45^+^) in liver after 2 weeks determined by flow cytometry (n=8). (f-g) Total liver lysates were analyzed by RNA sequencing after 3 weeks of NCD or SSD feeding (n=4-5). (f) Volcano plot of differentially expressed genes. (g) Expression of key genes associated with liver fibrosis. (h) LC-MS/MS analysis of lipid species in the liver and plasma of mice fed for 18 days with NCD, or SSD. (i) BODIPY staining of hepatocytes isolated from liver 2 weeks after initiation of SSD feeding (n=3). (j) Quantification of NKG2D ligand-expressing cells within BODIPY^Bright^ hepatocytes after 2 weeks of NCD or SSD feeding (n=3). (k) Quantification of the NKG2D ligand H60 by qPCR (n=3) normalized to HPRT expression. (l) Levels of cholesterol in liver lysate and plasma samples of NCD or SSD fed mice determined by LC-MS/MS analysis. Shown are representative data of at least two experiments, or pooled data of two independent experiments (a,b,d). Shown are means +/- s.e.m. Statistical significance was determined by unpaired t-test (a-j, l) and ANOVA with Bonferoni post-testing (k). Statistical significance was defined as *p<0.05; **p<0.01; ***p<0.001.

To gain mechanistic insight into early hepatic stress responses in the context of MAFLD, we established a mouse model for this disease. Current dietary models of MAFLD are typically based on the exclusion of key nutrients, such as choline and methionine, and are therefore not an ideal representation of human disease (25). We formulated a modified *Amylin liver NASH diet* (26), which we termed the steatosis-steatohepatitis diet (SSD). SSD contains high levels of fat, fructose and cholesterol and mimics an unhealthy Western lifestyle. SSD-fed mice rapidly gained body weight (Fig. 2a) and accumulated visceral adipose tissue mass (Fig. S2a), which is one of the hallmarks of metabolic syndrome. Compared to normal chow diet (NCD)-fed controls, SSD-fed animals displayed hepatomegaly and showed an increased liver to body weight ratio (Fig. 2b), a key feature of liver pathology in humans (27). We also observed an increase in the liver enzymes ALT and AST in the serum, which are clinical markers of liver damage (Fig. 2c).

Histological analysis of murine liver samples from SSD-fed mice showed a gradual progression of fatty liver disease, which was highly similar to the pathology seen in humans. After four weeks of feeding, we observed glycogen-induced distension of hepatocytes, followed by moderate micro- and macro-vesicular steatosis after eight weeks (Fig. S2b). As in human disease, pathology started in the periportal zone (zone I) and radiated outwards. At twelve weeks of SSD feeding, livers showed widespread steatosis with inflammatory foci, cyst formation, and hepatocyte degeneration, indicating a transition from MAFL to NASH (Fig. S2b). After 16 weeks, we could see advanced steatosis and considerable fibrosis in SSD-fed animals compared to NCD-fed controls, corresponding with NASH stage G3/F2 in humans (Fig. 1a and 2d). Additionally, a-smooth muscle actin (α-SMA) was strongly increased in SSD-fed mice, indicative of activated hepatic stellate cells (HSCs) and extracellular matrix deposition (Fig. 2d). Finally, in accordance with our histological findings, SSD feeding caused a progressive increase of immune cells in the liver. Surprisingly, this increase was already apparent two weeks after the initiation of feeding, suggesting that changes in the immunological micro-environment occur very early during development of NASH (Fig. 2e and Fig. S2c).

To elucidate the molecular changes underlying SSD-induced liver pathology early in the disease before apparent inflammation, RNA sequencing analysis was performed on total liver lysates of mice fed for three weeks with SSD. 1386 transcripts were more than two-fold differentially expressed between the groups, of which 969 were upregulated and 417 were downregulated in SSD-fed animals compared to NCD-fed controls (Fig. 2f). Amongst the pathways that were most affected by SSD feeding, several were associated with metabolism and inflammation, further confirming the validity of SSD feeding as a model for NASH (Fig. S2d,e). Finally, the analysis of individual transcripts showed that many putative genes associated with liver fibrosis such as *Acta2, Col1a1, Lox* and *Hhipl1* were upregulated in animals fed with SSD (Fig. 2g). Hepatic fibrosis was still observed in SSD-fed animals in which abdominal fat was surgically removed (Fig. S2f) indicating that the liver is the primary source of stress signals inducing NASH.

We considered that liver inflammation following SSD feeding is induced by hepatocyte death caused by accumulation of toxic lipid species. Therefore, we analyzed the lipid profile of livers of animals fed for three weeks with an SSD or NCD by mass spectrometry. We observed a significant increase of several lipid species previously associated with lipotoxicity, including ceramides and diacylglycerols (Fig. 2h and Table S1). However, levels of extracellular HMGB1, a marker associated with necrotic cell death, were not increased early after initiation of SSD feeding (Fig. S2g). In addition, granulomas, which mark sites of necrotic cell death, were not observed after four weeks of SSD feeding (Fig. S2h). Only after at least 8 to 16 weeks of SSD feeding, we could detect signs of inflammatory cell death (Fig. S2g,h). Thus, lipotoxicity does not appear to be a major cause of liver inflammation at early stages of MAFLD in the SSD model.

We next hypothesized that metabolic stress initiates liver inflammation through the activation of NKG2D. We measured NKG2D ligands on metabolically stressed hepatocytes. The lipid content of hepatocytes was visualized using BODIPY staining, and steatotic cells could be identified as BODIPY^High^ by flow cytometry (Fig. 2i). After 2 weeks of SSD feeding, steatotic cells showed significantly more expression of NKG2D ligands than hepatocytes isolated from NCD-fed controls by NKG2D-Fc staining (Fig. 2j). To determine which factor from the SSD diet drives NKG2D ligand expression, we generated cultures of primary mouse hepatocytes and loaded them for 48h with fructose, cholesterol, or oleic acid. Whereas oleic acid was most strikingly associated with lipid accumulation in hepatocytes, cholesterol was most effective at inducing NKG2D ligand expression, particularly of H60 (Fig. 2k and Fig. S2i,j).

Levels of cholesterol were highly increased in both the liver and plasma of SSD-fed mice (Fig. 2l). To demonstrate its importance for the development of hepatitis in vivo, we subjected mice to an SSD diet in which cholesterol was omitted. Indeed, this diet did not lead to inflammation *in vivo* (Fig. S2k). In addition, feeding of mice with a diet containing high levels of fat (HFD), an established model for obesity-induced visceral adipose tissue (VAT) inflammation and insulin resistance, resulted in obesity, hyperinsulinemia and glucose intolerance (28), but we detected only mild steatosis and no signs of liver fibrosis (Fig. S2l,m). Conversely, animals fed with a diet rich in cholesterol (HCD) did show immune cell accumulation in the liver (Fig. S2n). However, HCD-feeding did not cause steatosis or fibrosis (Fig. S2l,m), confirming previous observations (29) that the pathophysiology of NASH requires the cumulative impact of several metabolites.

In summary, SSD feeding is a valid model for human NASH and causes NKG2D ligand induction in hepatocytes following cholesterol accumulation.

### NKG2D engagement is essential for the development of liver fibrosis in the context of NASH

**Figure 3:**
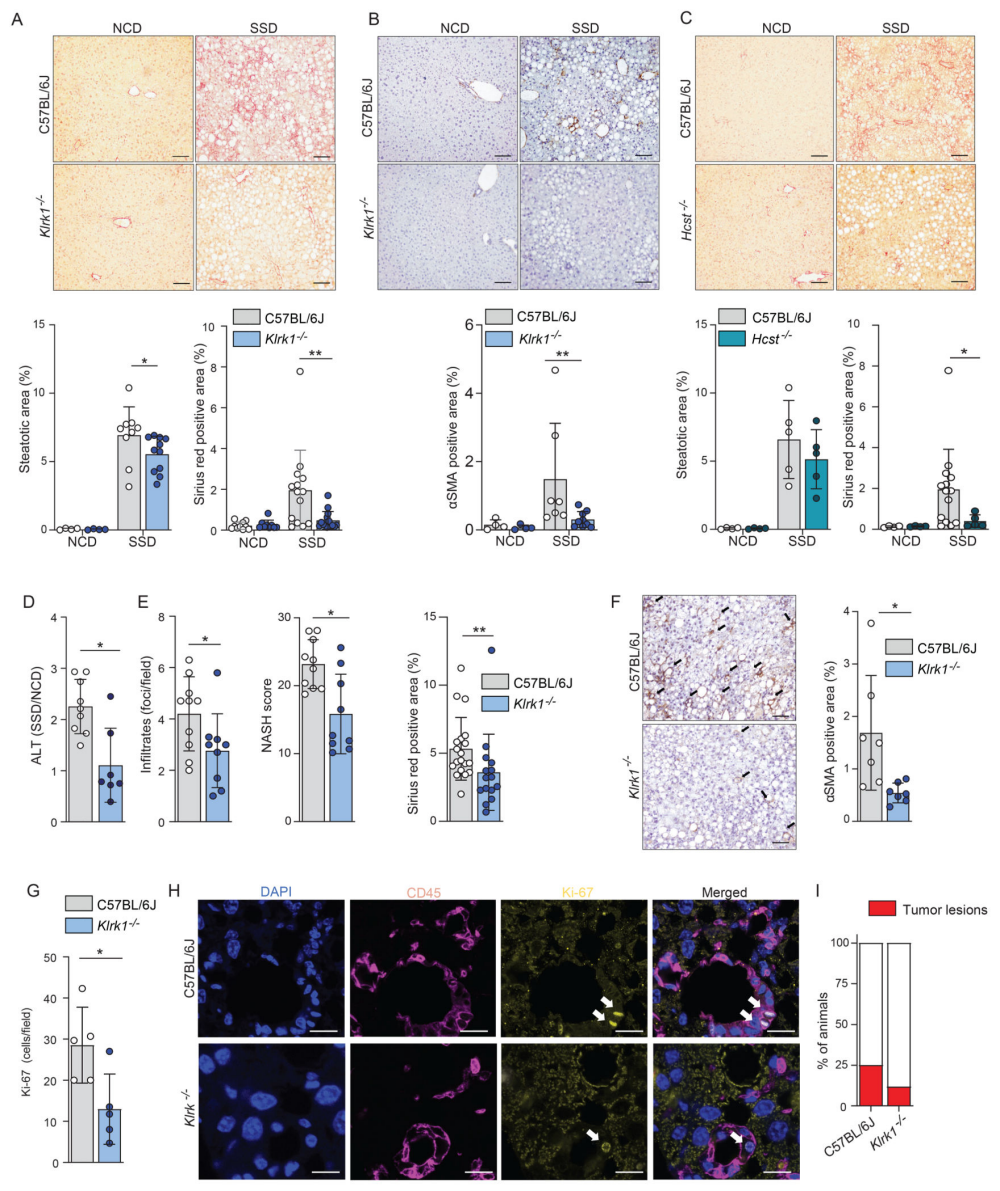
NKG2D engagement is essential for development of liver fibrosis in context of NASH. (a-b) WT and *Klrk1*^*-/-*^ (NKG2D-deficient) mice were fed an NCD or an SSD diet for 16 weeks (n=4-11). (a) (top) Representative liver slides stained with Sirius Red (200 X) and (bottom) quantification of steatosis and fibrosis. Scale bars indicate 100 µm. (b) (top) Representative liver slides stained for αSMA and (bottom) quantification of hepatic stellate cell activation (200 X). Scale bars indicate 100 µm. (c) WT and *Hcst*^-/-^ (DAP10-deficient) were fed an NCD or an SSD diet for 16 weeks. (top) Representative liver slides stained with Sirius Red (200 X) and (bottom) quantification of steatosis and fibrosis by Sirius red staining Scale bars indicate 100 µm. (n = 4-5). (d-i) WT and *Klrk1*^*-/-*^ mice were fed an NCD or an SSD diet for 52 weeks and livers were analyzed (n=7-9). (d) quantification of ALT in serum. (e) quantification of liver pathology of histological slides stained with H&E or Sirius red. (f) (left) Representative liver slides stained for αSMA and (right) quantification of hepatic stellate cell activation (200 X). Scale bars indicate 100 µm. (g) slides were stained for Ki67 and positive cells per field of view were quantified. (h) Representative immunofluorescence staining of CD45 and Ki67. Arrows mark Ki67^+^ nuclei. Scale bars indicate 10 µm. (i) number of mice carrying macroscopically visible tumors were quantified (n=17-20). The data are representative of at least two independent experiments or show pooled data of two experiments. Shown are means +/- s.e.m. Statistical significance was determined by unpaired t test. Statistical significance was defined as *p<0.05; **p<0.01; ***p<0.001.

To determine whether NKG2D signaling is essential for the induction of fibrosis in our model for NASH, we fed NKG2D-deficient (*Klrk1*^-/-^) mice (30) with an SSD diet and analyzed the level of collagen deposition after 16 weeks. We found a striking reduction in the level of fibrosis in livers of *Klrk1*^-/-^ mice compared to wild type C57BL/6J (WT) controls, whereas steatosis was not greatly affected (Fig. 3a). Immunohistological staining of aSMA also showed a strong reduction of its expression in *Klrk1*^-/-^ mice (Fig. 3b). In most immune cells, NKG2D uses the DAP10 adaptor molecule to mediate intracellular signaling (20). To confirm the importance of NKG2D signaling in NASH progression, we placed DAP10 deficient (*Hest*^-/-^) mice on an SSD. After 16 weeks, we compared the liver pathology in DAP10-deficient mice with that of SSD co-fed WT animals. The level of fibrosis and the aSMA-expression in livers of *Hcst*^*-/-*^ mice were both significantly reduced in comparison to WT mice. In contrast, levels of steatosis were not different between the SSD-fed groups of mice (Fig. 3c and Fig. S3a).

NASH strongly increases the overall risk of developing cirrhosis and hepatocellular carcinoma (HCC) (4). Previously, NKG2D-mediated inflammation was shown to promote development of HCC (31). We therefore investigated the impact of NKG2D-deficiency on long-term SSD feeding. WT and *Klrk1*^*-/-*^ mice were placed on SSD for one year before analysis of hepatic pathology. Liver damage was significantly reduced in *Klrk1*^*-/-*^ mice, as shown by much lower levels of ALT in circulation (Fig. 3d). Histochemical analysis revealed that virtually all parameters associated with NASH, including NASH score, fibrosis, αSMA induction, and leukocyte infiltrations were reduced in *Klrk1*^*-/-*^ mice compared to WT controls (Fig. 3e,f). Proliferation of cells, as determined by Ki67 staining, was predominantly observed within CD45^+^ immune cells and was significantly reduced in the livers of NKG2D-deficient animals (Fig. 3g,h), which is in line with reduced immune cell activation in this tissue. Finally, the incidence of tumors, which was approximately 25% in livers of one-year SSD fed WT animals, was more than two-fold reduced in the livers of *Klrk1*^*-/-*^ mice, as was the average tumor size at the time point of analysis (Fig. 3i and Fig. S3b, c).

Taken together, we conclude that NKG2D plays an important role in the development of NASH and long-term liver disease in the context of MAFLD.

### Liver fibrosis in the context of NASH is mediated by innate-like T cells

**Figure 4:**
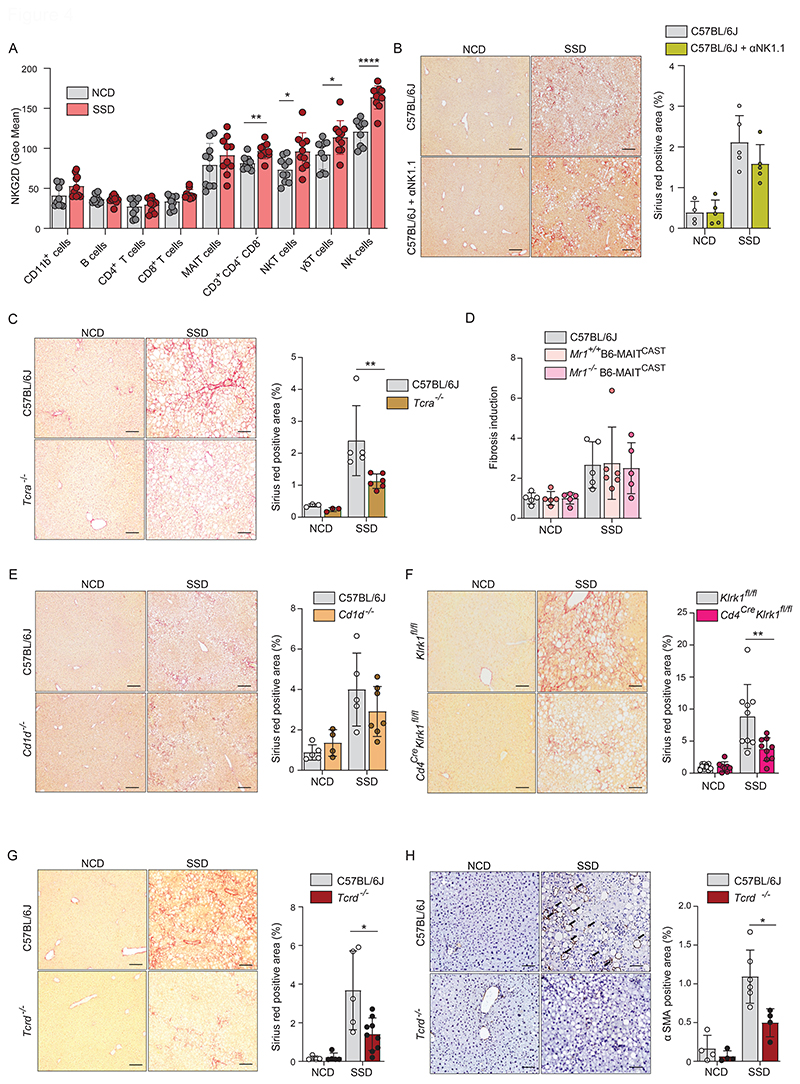
Liver fibrosis in the context of NASH is mediated by innate-like T cells. (a) WT mice were fed with an NCD or SSD for 18 days and NKG2D expression was quantified in key hepatic leukocyte populations by flow cytometry (n=5). (b) WT mice and C57BL/6J mice treated with NK cell-depleting antibody on a weekly basis were fed an NCD or an SSD diet for 16 weeks. Shown are (left) representative liver slides stained with Sirius Red (200 X) and (right) quantification of fibrosis (n = 4-5) (c) WT and *Tcra*^*-/-*^ (T cell receptor δ-chain deficient) mice were fed an NCD or an SSD diet for 16 weeks. Shown are (left) representative liver slides stained with Sirius Red (200 X) and (right) quantification of fibrosis (n = 5). (d) WT, *Mr1*^*+/+*^B6-MAIT^CAST^ and *Mr1*^*-/-*^B6-MAIT^CAST^ mice were fed an NCD or an SSD diet for 16 weeks. Quantification of fibrosis is shown (n = 5-6). (e) WT and *Cd1d*^*-/-*^ mice were fed an NCD or an SSD diet for 16 weeks. Shown are (left) representative liver slides stained with Sirius Red (200 X) and (right) quantification of fibrosis (n = 4-7). (f) *CD4*^*Cre*^*Klrk1*^*Flox/Flox*^ and *Klrk1*^*Flox/Flox*^ littermate controls were fed an NCD or an SSD diet for 16 weeks. Shown are (left) representative liver slides stained with Sirius Red (200 X) and (right) quantification of fibrosis (n = 9-10). (g, h) WT and *Tcrd*^*-/-*^ (T cell receptor α-chain deficient) mice were fed an NCD or an SSD diet for 16 weeks (n=4-10). (g) Shown are (left) representative liver slides stained with Sirius Red (200 X) and (right) quantification of fibrosis. (h) Shown are (left) representative liver slides stained for αSMA and (right) quantification of hepatic stellate cell activation (200 X). Scale bars indicate 100 µm. The data are representative of at least two independent experiments or pooled data of at least two experiments (a, f). Shown are means +/-s.e.m. Statistical significance was determined by unpaired t test. Statistical significance was defined as *p<0.05; **p<0.01; ***p<0.001.

NKG2D is expressed on various lymphocytes subsets. We therefore determined which immune cells in the liver express NKG2D and whether expression changes in response to SSD feeding. CD11b^+^ myeloid cells did not express NKG2D and expression was also very low on adaptive immune cells. In contrast, MAIT, CD3^+^CD4^-^CD8^-^ double negative (DN) T cells, NKT, γδ T cells and NK cells expressed high levels of NKG2D and for the latter four its expression was further increased in response to a NASH-inducing diet (Fig. 4a and Fig. S4a). Further analysis revealed that all NKG2D-expressing cells also increased in absolute numbers upon two weeks of SSD feeding, relative to NCD-fed controls (Fig. S4b).

To identify which of these cell populations contributes to the development of NKG2D-mediated NASH, we made use of several genetically modified mouse lines. First, we generated NK cell specific NKG2D-deficient mice by crossing *Ncr1*^*Cre*^ mice with *Klrk1*^fl/fl^ animals (32) and placed them on an SSD for 16 weeks. Histological analysis of liver slides showed that *Ncr1*^*cre*^*Klrk1*^fl/fl^ and *Klrk1*^fl/fl^ littermates had similar levels of liver fibrosis (Fig. S4c). Furthermore, NK cell depletion did not impair fibrosis development in SSD-fed mice, excluding a role for these cells in the development of NASH in this model (Fig. 4b).

Apart from NK cells, NKG2D was highly expressed on the TCRα^+^ subsets MAIT cells, NKT cells and CD4^-^CD8^-^ double-negative (DN) T cells in the liver. We therefore investigated the importance of these cells in the development of liver fibrosis. Mice with genetic deficiency for the α-chain of the TCR were placed on SSD, and collagen deposits were quantified after 16 weeks. We observed that fibrosis was significantly reduced in these animals, though not to the levels of NCD-fed animals (Fig. 4c). Steatosis was not affected in these mice, suggesting an impact on inflammation but not on hepatic metabolism (Fig. S4d). To determine which NKG2D^+^TCRα^+^ T cell subset mediates SSD-induced liver fibrosis, we first investigated the role of MAIT cells. We made use of MAIT^CAST^ mice, which contain approximately 10-fold more MAIT cells than WT controls. MAIT cells recognize antigen presented in the context of the non-classical MHC molecule MR1. Animals deficient for this molecule therefore do not have MAIT cells (33). WT, *Mr1*^*+/+*^ B6-MAIT^CAST^ and *Mr1*^*-/-*^ B6-MAIT^CAST^ animals were placed on an SSD and liver fibrosis was determined after 16 weeks. Neither the absence nor the increased presence of MAIT cells resulted in a change in the level of liver fibrosis (Fig. 4d). We therefore conclude that MAIT cells do not contribute to the development of fibrosis in the SSD model.

The vast majority of NKT cells in liver are CD1d-restricted (34). To investigate the role of NKT cells in NASH, *Cd1d*^*-/-*^ mice were placed on an SSD for 16 weeks and liver fibrosis was analyzed. We did not observe a significant reduction in the amount of fibrosis in these animals compared to WT controls (Fig. 4e). Thus, CD1d-restricted NKT cells do not play a role in the development of SSD-induced NASH. Finally, we wanted to investigate the role of DN T cells in the development of SSD-induced NASH. Unfortunately, the lack of cell-specific markers does not allow targeting of only this immune cell subset. However, all TCRα^+^ cells go through a CD4^+^ phase during thymic development. Since we excluded a role for MAIT and CD1d-restricted NKT cells in development of SSD-induced NASH, we therefore made use of Cd4^cre^*Klrk1*^fl/fl^mice. Cd4^cre^*Klrk1*^fl/fl^ mice and *Klrk1*^fl/fl^ littermates were placed on SSD and liver fibrosis was quantified after 16 weeks. We observed a significant reduction of fibrosis in the livers of *Cd4*^cre^*Klrk1*^fl/fl^ animals compared to *Klrk1*^fl/fl^ littermates, which was comparable to that observed in TCRα^-/-^ mice (Fig. 4c,f). Steatosis was not affected in these animals (Fig. S4e). Thus, our findings indicate that DN T cells are important for NKG2D-dependent liver fibrosis in the context of NASH.

Neither CD4^cre^*Klrk1*^fl/fl^ nor TCRα^-/-^ mice showed a complete abrogation of liver fibrosis following SSD feeding. We therefore hypothesized that γδ T cells also contribute to NKG2D-mediated liver fibrosis in our NASH model. Unfortunately, no model is available to specifically target genes in γδ T cells as the existent *Tcrd*^CreERt2^ model has very poor efficiency in the liver (35). Therefore, to determine the importance of γδ T cells in SSD-induced liver pathology, we made use of TCRδ^-/-^ mice, which lack all γδ T cells. After 16 weeks of SSD feeding, TCRδ^-/-^ animals showed a significant reduction in liver fibrosis compared to WT controls, whereas steatosis was not affected (Fig. 4g and Fig. S4f). In addition, α-SMA expression was significantly lower in the livers of SSD-fed TCRδ^-/-^ mice (Fig. 4h).

In summary, NKG2D-mediated liver fibrosis in context of MAFLD is mediated by the concerted action of DN αβ T cells and γδ T cells.

### NKG2D mediates liver fibrosis through IL-17A induction

**Figure 5:**
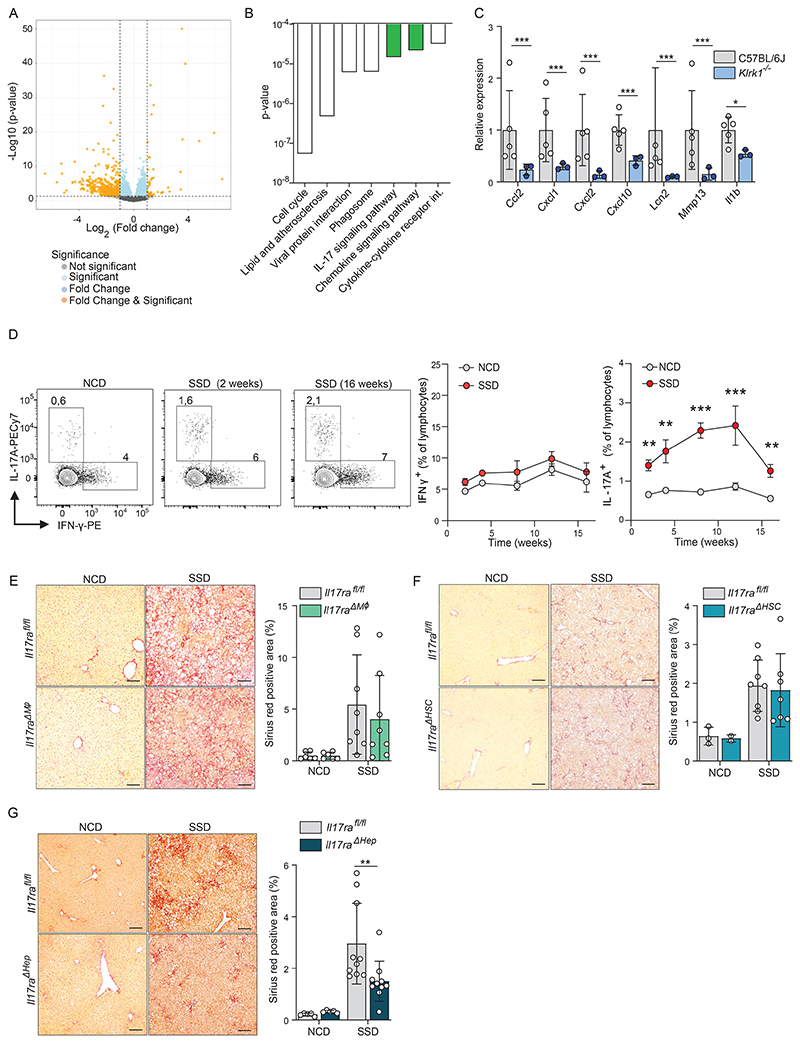
IL-17A signaling to hepatocytes drives liver fibrosis in the context of NASH. (a-c) WT and *Klrk1* ^*-/-*^ mice were fed an SSD for 3 weeks and the total transcriptome of liver tissue was analyzed by RNA sequencing. (a) Volcano plot of genes differentially expressed between WT and *Klrk1* ^*-/-*^ mice (n=3-5). (b) Most downregulated KEGG-pathways in *Klrk1*^*-/-*^ mice compared to WT controls by p-value. Marked in green are the IL-17A receptor signaling pathway and the chemokine signaling pathway. (c) Differential expression of downstream target genes of the IL-17A receptor signaling pathway. (d) WT mice were fed with an NCD or SSD diet. At indicated time points, liver leukocytes were restimulated *in vitro* with PMA/Ionomycin and cytokine production was measured by flow cytometry (n = 10). Representative plots are gated for lymphocytes. Numbers represent the percentage of IFN-*γ* or IL-17A-producing cells. (Right panel) Kinetics of hepatic IFNγ- and IL17A-producing lymphocytes over time. (e) *IL17ra^fl/fl^* and Lys *^Cre^IL7ra^fl/fl^* (*IL17ra*^*ΔMφ*^) littermates were fed an NCD or an SSD diet for 16 weeks. Shown are (left) representative liver slides stained with Sirius Red (200 X) and (right) quantification of fibrosis (n=8). (f) *IL17ra^fl/fl^* and GFAP*^Cre^IL17ra^fl/fl^* (*IL17ra*^*ΔHSC*^) littermates were fed an NCD or an SSD diet for 16 weeks. Shown are (left) representative liver slides stained with Sirius Red (200 X) and (right) quantification of fibrosis (n = 2-7). (g) *IL17ra^fl/fl^* and Albumin*^Cre^ IL17ra^fl/fl^* (*IL17ra*^*ΔHep*^) littermates were fed an NCD or an SSD diet for 16 weeks. Shown are (left) representative liver slides stained with Sirius Red (200 X) and (right) quantification of fibrosis (n = 5). Scale bars indicate 100 µm (e-g). The data are representative of at least two independent experiments or pooled data of at least two experiments (d-g). Shown are means +/-s.e.m. Statistical significance was determined by unpaired t test. Statistical significance was defined as *p<0.05; **p<0.01; ***p<0.001.

NKG2D can mediate both cytotoxicity and cytokine production of NK cells and innate-like T cells (ILTs) (20, 36). We therefore investigated which signaling pathways are most affected by NKG2D deficiency early after initiation of metabolic stress induced by SSD feeding. RNA sequencing of total liver lysates revealed 496 transcripts that were differentially expressed at least 2-fold between WT and *Klrk1*^*-/-*^ mice after 3 weeks of SSD feeding. Of these, 39 showed higher and 457 showed lower expression in NKG2D-deficient animals (Fig. 5a). When we performed a KEGG-pathway analysis on differentially expressed genes, we observed that the IL-17A receptor and the chemokine signaling pathways were amongst the most negatively affected in *Klrk1*^*-/-*^ animals (Fig. 5b). Indeed, analysis of individual genes reveled that several transcriptional targets of the IL-17A receptor signaling pathway, including chemokines such as *Cxcl1, Cxcl2* and *Ccl2*, were strongly reduced in SSD-fed NKG2D-deficient animals compared to WT controls (Fig. 5c). Inflammatory pathways were not affected in NCD-fed *Klrk1*^*-/-*^ mice compared to WT controls (Fig. S5a,b). We therefore hypothesized that NKG2D-dependent induction of IL-17A expression mediates liver fibrosis in the context of MAFLD. To confirm this, we first determined the importance of IL-17A in the pathology of SSD-induced NASH. Analysis of cytokine expression by hepatic lymphocytes showed that IL-17A levels were rapidly and strongly increased after initiation of SSD feeding and stayed high for the duration of the experiment. In contrast, expression of IFNγ and TNF was not affected by SSD-induced metabolic stress (Fig. 5d and Fig. S5c). IL-17A levels did not increase after HFD feeding, indicating that this cytokine may be specifically involved in the induction of liver fibrosis (Fig. S5d).

IL-17A is important for several homeostatic processes, including the maintenance of gut barrier integrity and adipose tissue metabolism (37, 38). We therefore made use of *IL17ra*^*fl/fl*^ mice to abrogate IL-17A sensitivity of specific cells. First, we crossed *IL17ra*^*fl/fl*^ mice with lysozyme^Cre^ (*Lys*^*Cre*^) animals to eliminate the IL-17A receptor in macrophages (*IL17ra*^*ΔMφ*^). However, *IL17ra*^*ΔMφ*^ mice showed a similar level of fibrosis after 16 weeks of SSD feeding as *IL17ra*^*fl/fl*^ littermate controls, indicating that IL-17A does not directly target these cells (Fig. 5e). IL-17A was shown to directly activate HSCs in a mouse model of NASH (14). We therefore crossed *IL17ra*^*fl/fl*^ mice with GFAP^Cre^ animals, which eliminates the IL-17A receptor from hepatic stellate cells and cholangiocytes (39). 16 weeks of SSD feeding did not show differences in liver fibrosis between *IL17ra*^*ΔHSC*^ mice and littermate controls (Fig. 5f). Finally, we questioned whether IL-17A might directly target hepatocytes. To demonstrate this, *IL17ra*^*fl/fl*^ mice were crossed with albumin^Cre^ (*Alb*^*Cre*^) animals to generate *IL17ra*^*ΔHep*^ mice. Indeed, after 16 weeks of SSD feeding, we observed a significant reduction in the amount of fibrosis in livers of animals with hepatocyte-specific deficiency for the IL-17A receptor compared to littermate controls (Fig. 5g). The level of steatosis was comparable between groups, indicating an impact on inflammation rather than on hepatic metabolism (Fig. S5e).

In summary, metabolic stress of the liver leads to increased expression of IL-17A by leukocytes in this organ, which directly targets hepatocytes.

### NKG2D stimulation promotes IL-17A production by hepatic γδ T cells in the context of NASH

**Figure 6:**
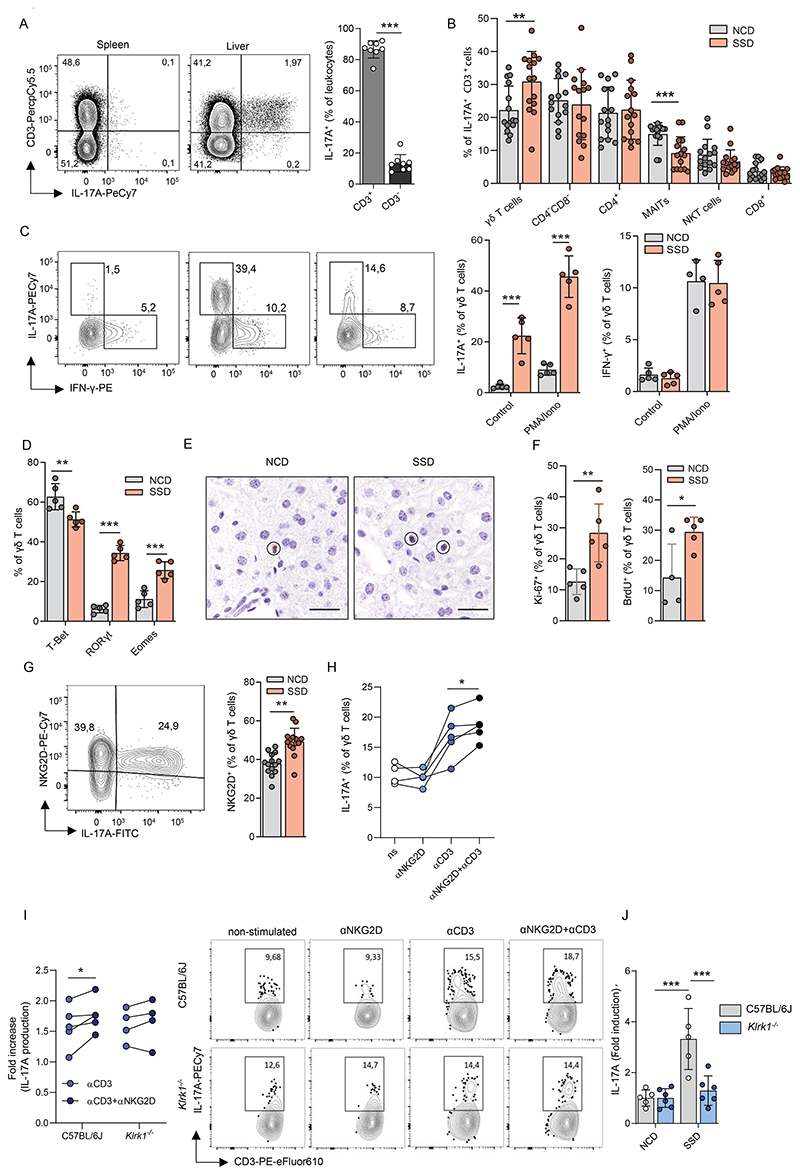
Liver fibrosis in context of NASH is mediated by innate-like T cells (a-b) WT mice were fed with an NCD or SSD diet and after 18 days liver leukocytes were restimulated *in vitro* with PMA/Ionomycin and IL-17A production was quantified by flow cytometry. (a) Fraction of CD3^+^ and CD3^-^ cells within the IL-17A^+^ cell pool (n = 8). Representative plots are gated for lymphocytes. (b) Indicated immune cell subsets were quantified within the CD3^+^IL-17A^+^ cell pool (n=15). (c) IL-17A and IFN-γ production by hepatic γδ T cells upon *in vitro* re-stimulation with PMA and ionomycin in WT mice after 2 weeks of NCD or SSD feeding (*n* = 5). Representative FACS plots are gated for γδ T cells. The number next to the outlined area represents the percentage of IFN-*γ*- or IL-17A-producing γδ T cells. (d) WT mice were fed an NCD or an SSD diet for 2 weeks and liver resident γδ T cells were analyzed by flow cytometry for expression of transcription factors (n=5). (e) CD3ε staining of *Tcra*^*-/-*^ mice after 2 weeks of feeding with NCD or SSD. Scale bars indicate 100 µm. (f) WT mice were fed an NCD or SSD for 2 weeks and for the last 4 days given BrdU in drinking water. Tissue-resident cells were visualized by injecting animals with biotinylated CD45 five minutes before isolation of livers, followed by fluorescent labeling using streptavidin-eFluor780 and analysis by flow cytometry. Tissue-resident cells were defined as eFluor-780^-^. Ki67^+^ and BrdU^+^ hepatic γδ T cells were quantified by flow cytometry (n = 5). (g) Quantification of NKG2D expression on hepatic γδ T cells by flow cytometry of mice fed two weeks with an NCD or SSD (n=14). The representative plot shows γδ T cells after PMA/Ionomycin stimulation. (h-i) Mice were fed an SSD for 2 weeks. Hepatic γδ T cells were restimulated *in vitro* and IL-17A production was determined by flow cytometry. (h) IL-17A production of γδ T cells after stimulation with αCD3, plate-bound αNKG2D or their combination, measured by flow cytometry. (i) Relative increase of IL-17A production within hepatic γδ T cells from WT or *Klrk1*^*-/-*^ mice upon *in vitro* re-stimulation with αCD3 alone or in combination with plate-bound αNKG2D over non-stimulated γδ T cells (*n* = 5). Representative FACS plots are gated for γδ T cells. Numbers show percentages of IL-17A-producing γδ T cells (j) WT and Klrk1^-/-^ mice were fed an SSD for 2 weeks. Hepatic γδ T cells were restimulated *in vitro* with PMA/Ionomycin and IL-17A production was measured by flow cytometry. Shown is the relative increase in IL-17A production within SSD-fed over NCD-fed mice (n=5-6). The data are representative of at least two independent experiments. Shown are means +/- s.e.m. Statistical significance was determined by unpaired t-test (a-g), paired t-test (h-i) and ANOVA with Bonferoni post-testing (j). Statistical significance was defined as *p<0.05; **p<0.01; ***p<0.001.

Our findings indicate that in the context of MAFLD, γδ T cells and a subset of TCRα^+^ cells are responsible for the induction of liver fibrosis in response to NKG2D stimulation. We therefore investigated which of these cells produce IL-17A early after initiation of SSD feeding. *In vitro* restimulation of hepatic leukocytes isolated from the liver two weeks after initiation of SSD feeding showed that IL-17A is produced almost exclusively by T cells (Fig. 6a). Further analysis of immune cell subsets within the IL-17A^+^ population indicated that this cytokine is predominantly secreted by CD4^+^ T_H_17, CD3^+^CD4^-^CD8^-^ T cells and γδ T cells. Of these, only IL-17A producing γδ T cells increased significantly upon SSD feeding (Fig. 6b and Fig. S6a). NKT cells were not a major source of IL-17A in our model, suggesting that γδ T, and to a lesser extent CD4^-^ CD8^-^ T cells, play a dominant role in the induction of NKG2D-mediated fibrosis following SSD feeding.

γδ T cells increased after SSD-but not HFD-feeding and showed the strongest increase in IL-17A production (Fig. 6b and Fig. S6b). We therefore decided to focus on the role of γδ T cells in NKG2D-dependent IL-17A production in the liver. Following SSD feeding, hepatic γδ T cells rapidly and potently induced IL-17A production, whereas their ability to produce IFNγ, TNF, IL-17F and IL-22 was not affected (Fig. 6c and Fig. S6c,d). In accordance with this cytokine profile, γδ T cells induced the expression of the transcription factors RORyt and Eomes, whereas there was a relative reduction in the percentage of cells expressing T-bet (Fig. 6d). Multi-dimensional analysis of γδ T cell populations by t-distributed stochastic neighbor embedding (t-SNE) revealed that animals fed with an NCD contained a relatively homogenous population with regards to CD44 and CD27 expression. In contrast, SSD feeding induced polarization of γδ T cells towards an IL-17A^+^CD27^-^CD44^Bright^ profile (Fig. S6e), a phenotype typically associated with IL-17A producing γδ T cells (γδ^17^ T cells) (40). Moreover, in SSD fed mice, IL-17A was predominantly produced by Vγ6^+^ cells and was associated with a relative decrease in the frequency of Vγ1^+^ cells within the γδ T cell pool compared to NCD-fed controls (Fig. S6f).

To confirm that γδ^17^ T cells play a general role in development of NASH pathology and not just in the SSD model, animals were fed with a methionine and choline deficient diet (MCD). This model induces liver steatosis and fibrosis after 12 weeks of feeding due to an inability of hepatocytes to form very-low-density lipoproteins, although it causes weight loss rather than weight gain in animals (41). WT and *Tcrd*^*-/-*^ mice were placed on an MCD, and immune cells and liver pathology were analyzed after 12 weeks. MCD caused an increase in γδ T cells in livers of WT animals, which was comparable to that observed after SSD feeding (Fig. S6g). γδ T cells significantly increased IL-17A production and became the dominant source of this cytokine in the liver (Fig. S6h,i). Notably, MCD-fed *Tcrd*^*-/-*^ mice had reduced pathology compared to WT controls, as they lost significantly less weight (Fig. S6j). In addition, liver fibrosis was considerably lower in *Tcrd*^*-/-*^ mice (Fig. S6k).

Previously, a lipid-rich environment was shown to promote the specific outgrowth of γδ^17^ T cells, as these cells preferentially use oxidative phosphorylation to fulfill their energetic needs (42). We therefore hypothesized that the increase of γδ T cells in steatotic livers was the result of local proliferation. We observed that γδ T cells predominantly resided in the parenchyma of the liver and not in the sinusoids, both after NCD and SSD-feeding (Fig. 6e). In vivo labeling of CD45^+^ cells in the circulation of SSD fed mice confirmed that a larger fraction of γδ T cells was tissue-resident in the liver than αβ T cells, explaining why the former population more vigorously responds to NKG2D ligands expressed on hepatocytes (Fig S6l). To exclude increased influx from the periphery as a reason for γδ T cell increase, we isolated leukocytes from the livers of animals two weeks after initiation of SSD-feeding and injected them in NCD or SSD co-fed animals. We did not observe a difference in the number of either αβ or γδ donor T cells infiltrating the spleen or liver of NCD or SSD-fed mice (Fig. S6m). We therefore analyzed proliferation of hepatic, tissue-resident γδ T cells in response to two weeks of SSD feeding and found that their expression of Ki67 was significantly increased (Fig. 6f). In addition, SSD-fed animals pulsed with BrdU showed a significant increase of its incorporation in hepatic γδ T cells (Fig. 6f). Importantly, cells expressing NKG2D and RORγT proliferated faster than γδ T cells not expressing these proteins as shown by Ki67 staining (Fig. S6n,o). These results suggest that the increase of NKG2D^+^ γδ^17^ T cells in livers of SSD-fed mice is due to preferential outgrowth of tissue-resident cells rather than ingress from the periphery.

Finally, we wanted to determine whether NKG2D stimulation of hepatic γδ T cells indeed promotes their production of IL-17A. We found that virtually all γδ T cells that produce IL-17A also express NKG2D on their surface. Moreover, the total fraction of NKG2D-expressing γδ T cells in the liver significantly increased in response to SSD-feeding (Fig. 6g). Next, we stimulated γδ T cells *in vitro* with agonistic antibodies against NKG2D, CD3ε or both simultaneously. We observed that NKG2D only promoted IL-17A production when stimulated in concert with the TCR (Fig. 6h,i). Importantly, this synergistic effect was not observed in γδ T cells derived from *Klrk1*^*-/-*^ mice, indicating that NKG2D potentiates a TCR-mediated signal to drive cytokine production (Fig. 6i). To confirm these observations *in vivo*, WT and *Klrk1*^*-/-*^ mice were placed on an NCD or SSD for two weeks, and IL-17A production was measured after *in vitro* restimulation of hepatic γδ T cells with PMA/Ionomycin. Whereas WT cells showed a strong increase in the number of γδ T cells producing IL-17A upon SSD feeding, *Klrk1*^*-/-*^ mice showed no differences in cytokine output (Fig. 6j). The absolute number of γδ T cells in the liver was not affected by NKG2D deficiency (Fig. S6p). Thus, NKG2D stimulates IL-17A production by tissue resident γδ T cells in context of MAFLD.

### NKG2D-signaling drives inflammation in the context of NASH

**Figure 7:**
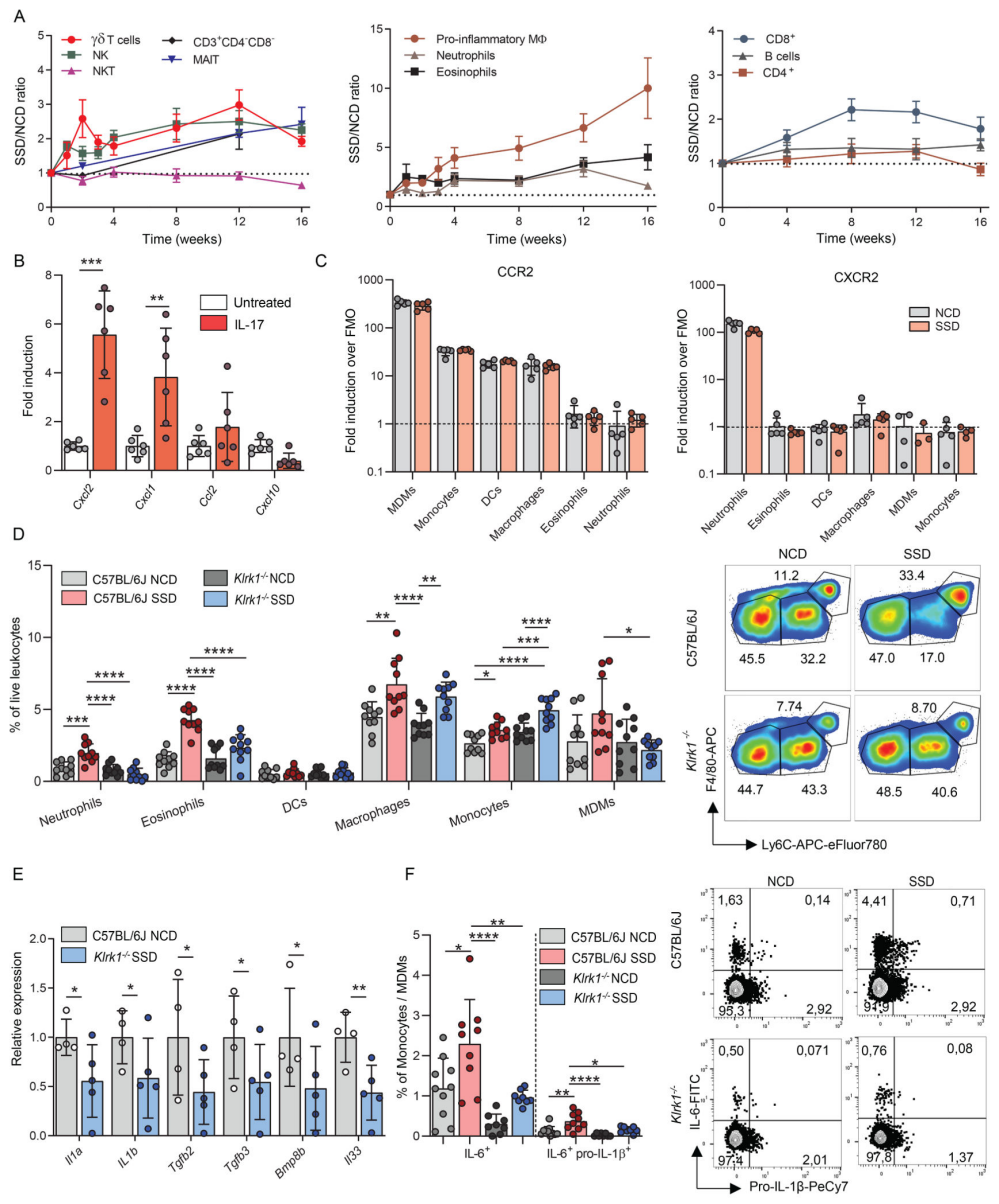
NKG2D mediates myeloid cell recruitment in the context of NASH. (a) WT mice were fed with an NCD or SSD and at the indicated times hepatic immune cell numbers were quantified by flow cytometry (n=6-10). (b) primary hepatocytes were cultured with or without IL-17A and expression of indicated chemokines was determined by qPCR (n=6). (c) WT mice were fed an NCD or SSD for 4 weeks and the levels of CCR2 and CXCR2 expression on indicated myeloid cell populations were measured by flow cytometry. (d) WT and *Klrk1*^*-/-*^ mice were fed for 4 weeks with an NCD or SSD. Hepatic leukocytes of the myeloid lineage were quantified by flow cytometry (n=10). Representative plots are gated for CD11b^+^F4/80^+^ cells. (e) WT and *Klrk1* ^*-/-*^ mice were fed an SSD for 3 weeks and the total transcriptome of liver tissue was analyzed by RNA sequencing. Expression of indicated genes associated with inflammation (n=3-5). (f) WT and *Klrk1*^*-/-*^ mice were fed an SSD for 4 weeks and hepatic leukocytes were restimulated *in vitro* with PMA/Ionomycin for 4h. Production of indicated cytokines was measured by flow cytometry (n=9-10). Representative FACS plots are gated for monocyte/MDM populations. Numbers represent the percentage of cytokine-producing cells. The data are representative of one experiment using 3-5 biological replicates (e), two pooled experiments (b,d,f) or at least two independent experiments (a,c). Shown are means +/- s.e.m. Statistical significance was determined by ANOVA with Bonferoni post-testing (d,f) or by unpaired t test (a,c,e). Statistical significance was defined as *p<0.05; **p<0.01; ***p<0.001. DCs – Dendritic cells, MDMs – Monocyte-derived macrophages

To gain more insight into the inflammatory processes mediated by NKG2D during development of NASH, we first analyzed the kinetics of hepatic immune cell populations in response to SSD feeding. We noticed that the NASH-inducing diet caused an increase of immune cells in three waves. Within two weeks after initiating feeding, NK cells and γδ T cells increased and remained higher during the entire 16-week period of feeding (Fig. 7a). The numbers of NKT cells remained comparable between SSD- and NCD-fed mice in the entire experiment. Approximately four weeks after the initiation of feeding, we observed a second wave of immune cells infiltrating the liver, dominated by F4/80^Bright^, pro-inflammatory macrophages, and to a lesser extent, neutrophils and eosinophils (Fig. 7a). Third, at around 8 weeks of feeding, CD8^+^ T cell numbers increased, which agrees with other murine models for NASH (5). CD4^+^ T cell and B cell populations remained mostly unaffected by SSD feeding (Fig. 7a). These findings suggest that SSD-induced liver inflammation is initiated by a wave of innate-like lymphocytes, which drives recruitment of myeloid cells and ultimately promotes the accumulation of CD8 T cells.

Our RNA sequencing data indicated that IL-17A promotes chemokine production by liver cells (Fig. 5c), which may explain how innate-like lymphocytes initiate the second wave of myeloid cells. To confirm this finding, primary hepatocytes were stimulated *in vitro* with IL-17A, and chemokine expression was determined by qPCR. We observed that in response to stimulation, hepatocytes induced expression of the chemokines CXCL1 and CXCL2 and to a lesser extent CCL2 (Fig. 7b), which are potent recruiters of pro-inflammatory cells of the myeloid lineage (43). CXCR2 is the receptor for CXCL1 and CXCL2, whereas CCR2 is the receptor for CCL2. We observed that CXCR2 was highly expressed on hepatic neutrophils, whereas CCR2 could be detected on monocyte-derived macrophages (MDM), monocytes, and macrophages, independent of the diet (Fig. 7c). Most lymphocytes did not express either receptor (Fig. S7a). In accordance with the expression profile of these two chemokine receptors, we observed that the absolute number and percentage of neutrophils and Ly6C^+^ monocytes/macrophages increased in response to SSD-feeding (Fig. 7d and Fig. S7b-d). Importantly, the increase of most of these populations in response to SSD feeding was significantly less in animals deficient for NKG2D (Fig. 7d and Fig. S7c,d).

Finally, we analyzed whether the inflammatory environment was affected by NKG2D deficiency. Total transcriptome analysis of total liver lysates of WT and *Klrk1*^*-/-*^ mice after three weeks of SSD feeding showed a marked decrease in genes associated with IL-6 regulation (Fig. S7e), a cytokine that has been associated extensively with NASH (44). In addition, gene expression of several pro-inflammatory cytokines associated with NASH, including IL-1β, were significantly reduced in the livers of SSD-fed *Klrk1*^*-/-*^ animals compared to WT controls (Fig. 7e). We therefore analyzed whether NKG2D deficiency impacted production of these pro-inflammatory cytokines. Indeed, SSD-feeding resulted in a significant increase of both IL-6 and IL-1β production by monocytes and monocyte-derived macrophages. Importantly, production of these cytokines was significantly reduced in animals deficient for NKG2D (Fig. 7f and Fig. S7e).

In summary, NKG2D signaling is required for liver inflammation in the context of MAFLD and promotes pro-inflammatory cytokine production by myeloid cells.

### IL-17A^+^Rorγt^+^ γδ T cells in blood are a non-invasive biomarker for human MAFLD

**Figure 8:**
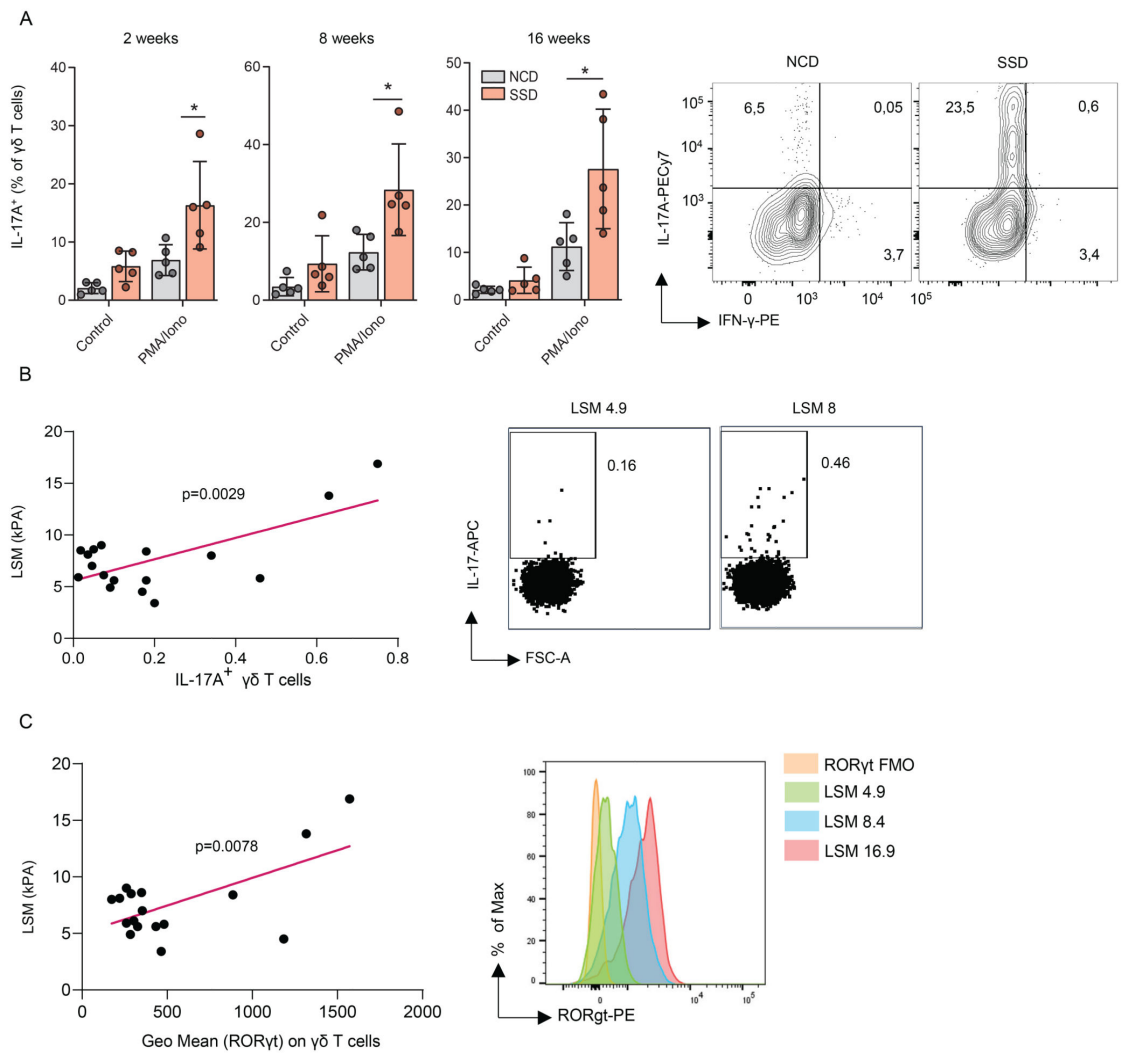
IL-17A^+^Roryt^+^ γδ T cells in blood are a potential non-invasive biomarker for human NASH (a) WT mice were fed with a NCD or SSD diet. At indicated time points, blood leukocytes were restimulated *in vitro* with PMA/Ionomycin and IL-17A production by γδ T cells was measured by flow cytometry (n=5). (b-c) human patients with MAFLD were analyzed for liver stiffness (LSM) by ultrasound measurement. Next, their peripheral blood mononuclear cells were restimulated *in vitro* with PMA/Ionomycin and γδ T cells were analyzed by flow cytometry. (b) correlation between liver stiffness measurement (LSM; an indicator of fibrosis) and IL-17A production by γδ T cells (n=18). (c) correlation between LSM and RORγt expression by γδ T cells (n=18). Shown are means +/- s.e.m. Statistical significance was determined by unpaired t-test (a), or by linear regression (b,c). Statistical significance was defined as *p<0.05; **p<0.01; ***p<0.001.

Few reliable biomarkers for NASH are currently available, especially during the early phases of the disease. The gold standard for diagnosis is a liver biopsy, which is an invasive procedure with a high chance of complications (4). We wanted to investigate whether changes in the immunological profile of hepatic immune cells associated with NASH can be detected in the blood. Mice were placed on an SSD and IL-17A production by γδ T cells isolated from the blood was determined after *in vitro* restimulation. We observed a significant increase in IL-17A producing γδ T cells in the blood 2 weeks after initiation of feeding, which closely mirrored their phenotype in the liver (Fig. 8a and Fig. S8a). In contrast, IFNγ and TNF production were not affected in these cells (Fig. S8a). The increased IL-17A production by γδ T cells in the blood was retained over a period of 16 weeks of SSD-feeding (Fig. 8a), indicating that it is a potential marker for metabolic stress in the liver in context of NASH.

To determine whether a similar profile could be detected in humans, blood was isolated from patients in whom MAFLD was confirmed by ultrasound techniques. Indeed, we observed a significant increase in IL-17A production by blood γδ T cells. This value positively correlated with the severity of liver stiffness (LSM; Fig 8b), which is a surrogate marker of fibrosis (45). A similar correlation was observed between liver stiffness and γδ T cells expressing the transcription factor Rorγt (Fig. 8c). In contrast, LSM did not correlate with either the total frequency of γδ T cells, or with the frequency of Th17 cells and IL-17A producing MAIT cells in the blood of patients (Fig. S8b,c). Even though they are not the dominant source of cytokines in circulation, these findings indicate that IL-17A production by γδ T cells best represents the inflammatory status of liver inflammation measurable in blood and has the potential to be a reliable non-invasive biomarker for NASH in humans.

In summary, metabolic stress of liver cells is sensed by tissue-resident innate-like T cells such as γδ T cells through the NKG2D receptor. In response, they produce IL-17A, which licenses hepatocytes to produce chemokines that recruit pro-inflammatory cells and mediate inflammation and fibrosis (Fig. S8d).

## Discussion

MAFLD affects approximately one quarter of the global adult population, yet its impact on public health has long remained unrecognized (46). The transition of MAFL to NASH marks the initiation of a process that may lead to fibrosis, loss of liver function and death (4). Elucidation of how this process is mediated is therefore of great clinical importance. Here we find that hepatocytes upregulate surface expression of NKG2D ligands following lipid accumulation. This signal of metabolic stress is detected by tissue-resident innate-like T cells (ILTs) and drives their production of IL-17A. Hepatocytes are licensed directly by IL-17A to produce chemokines that recruit pro-inflammatory cells into the liver, leading to development of NASH and fibrosis. Deficiency of NKG2D, γδ T cells, or the IL-17A receptor on hepatocytes could prevent inflammation and fibrosis while not impacting steatosis.

The transition of MAFL to NASH was proposed to require multiple instigators, such as oxidative stress, fat accumulation, and a microbiome imbalance (2). How these factors are translated into a signal that activates the immune system was unclear. Previously, microbiota-derived lipid antigens were shown to be important for MAFLD-induced expansion of γδ T cells in the liver, yet how these cells were subsequently activated was unclear (12, 14). Our findings indicate that lipid accumulation, most notably of cholesterol, leads to induction of NKG2D ligands and activation of γδ T cells. How cholesterol drives metabolic stress is unknown but may involve Liver X receptors (LXRs). LXRs are transcription factors that are activated in response to high cholesterol levels and mediate oxysterol metabolism (47). Agonists of these receptors were shown to induce expression of the NKG2D ligands MICA and MICB in human cells (47). Notably, reverse agonists of LXRs have a beneficial impact on systemic lipid metabolism and liver pathology in context of MAFLD and several of these compounds have been tested in clinical trials (48, 49). We cannot exclude that SSD feeding mediates activation of immune cells at other sites such as adipose tissue, which was shown to mediate IL-17A production in tissue-resident γδ T cells (38). Nevertheless, our finding that IL-17A receptor deficiency on hepatocytes does ameliorate pathology suggests that this cytokine primarily mediates its effect on the liver.

A hallmark of MAFLD is the formation of IL-17A-mediated type 3 inflammation, which is a driving force behind the pathogenesis of NASH and HCC (8, 13, 50–54). A recent study showed that the steatotic liver microenvironment gives rise to pro-inflammatory CXCR3^+^ Th17 cells which produce elevated levels of IL-17A and TNFα (55). However, Th17 cells are typically only formed later during the immune response and the signal that initiates immune polarization in MAFLD was left unexplored. Our findings indicate that in MAFLD, NKG2D ligands are the primary signal that communicates loss of tissue homeostasis to intra-parenchymal ILTs, which produce IL-17A to initiate type-3 inflammation. IL-17A predominantly targets non-hematopoietic cells including hepatocytes (43, 58). Interestingly, IL-17A receptor deficiency on hepatocytes did not protect animals from developing fibrosis to the extent seen in *Klrk1*^*-/-*^ mice, suggesting that additional factors are induced by NKG2D engagement. Recently, GM-CSF was shown to be important for development of fibrosis in animal models of NASH and γδ T cells can produce high levels of this cytokine (13, 59). Whether γδ T cell derived GM-CSF is also important for development of SSD-induced liver fibrosis is currently unclear. Nevertheless, production of chemokines that attract myeloid cells to the infected or injured site is the hallmark of IL-17A signalling (43, 60). Deficiency of this cytokine or its signalling components results in decreased hepatic accumulation of myeloid cells in models of NASH (7, 52). Our findings indicate that NKG2D-induced IL-17A production directly licenses hepatocytes to produce CXCL1, CXCL2 and CCL2, which were previously shown to be crucial for neutrophil accumulation, HSC activation and fibrosis in NASH (61, 62). Thus, our study elucidates the first step in metabolic stress-induced type-3 liver inflammation.

NKG2D is classically associated with Type-I immune responses against viruses and tumors, whereas we now show that this receptor drives IL-17A production. NKG2D is expressed on NK cells, antigen experienced CD8 T cells and NKT cells and their engagement of its ligands leads to the production of IFNγ, an archetype Th1 cytokine (20, 21, 36). These immune cells are also present in the liver and it was therefore surprising that NKG2D ligand induction by hepatocytes only activates a subset of ILTs specialized in the production of IL-17A. However, Th1- and Th17-type NKG2D^+^ immune cells are found at different sites in the liver. NK cells and NKT cells are predominantly present in the liver sinusoids and will therefore most efficiently respond to incursions such as viral infections which target the endothelium of the liver blood vessels and cells located within the space of Diss (63). Our findings indicate that hepatic γδ T cells are present in the parenchyma of the liver and are therefore the first responder to signals coming from hepatocytes. Indeed, also in human tissues most IL-17A^+^ cells of patients with NASH appeared to be in direct contact with hepatocytes. Thus, the location of NKG2D ligands appears crucial for the way in which the consequent immune response polarizes.

In summary, our study identifies a key mechanism for the transition from MAFL to NASH in patients with metabolic liver disease and has great potential as a therapeutic target for the treatment of MAFLD. In addition, the frequency of IL-17^+^RORγt^+^ γδ T cells in the blood of MAFLD patients positively correlated with ultrasound-based techniques for the quantification of fibrosis. Measurement of these cells in people with MAFLD therefore has great potential as a non-invasive marker for the diagnosis of NASH.

## Materials & Methods

### Study design

The goal of this study was to identify the mechanism via which hepatocytes communicate metabolic stress to the immune system and initiate the inflammation that marks the transition of MAFL to NASH. To this end, we generated a new dietary model to induce NASH in mice and analyzed changes in immune cell subsets following feeding. Sample size was determined by a power analysis based on previous experiments pilot studies. We assumed a power of 85%, a within group variation of 20-30% and between-group difference of 50-100%. A 95% confidence interval was considered statistically significant. Dependent on the parameter, group sizes per experiment were 4-8 animals. A similar power analysis was performed for experiments using human samples, which dictated the use of at least 16 samples. All data that was collected was also included in the analysis. Each experiment was performed at least twice under the same conditions. Experiments were blinded as much as possible through assignment of groups by people that were not further involved in the experiments.

### Patients

Patients were included at the NAFLD polyclinics at KBC Rijeka. All patients were over 18 years of age and signed an informed consent before inclusion. All patients were subjected to laboratory analysis, abdominal ultrasound (US), Transient Elastography (TE) measurements using a FibroScan 502 Touch (Echosense, Paris, France). Patients with incomplete data, those who refused to undergo TE or US examination, those with frequent alcohol consumption (>20g per day for men and >10 g per day for women), other chronic liver diseases (viral, metabolic, or autoimmune), celiac disease, and those with secondary causes of fatty liver such as drugs (amiodarone and tamoxifen) were excluded from the final analysis. Additionally, active malignancy, congestive heart failure and valvular heart disease, TE failure, and pregnancy were additional exclusion criteria.

In all patients TE examination after overnight fasting was done by FibroScan, which was performed using M or XL probe by an experienced gastroenterologist. The examination was defined as valid if there were ≥10 valid measurements with interquartile range- (IQR-) to-median ratio of LSM ≤0.3. The diagnosis of liver steatosis was considered in patients with CAP ≥ 238 dB/m (70). Patients with LSM ≥7 kPa were defined to have a high liver fibrosis (≥F2), while an advanced fibrosis (≥F3) was considered if LSM was ≥9.6 kPa using the M probe or ≥9.3 kPa using the XL probe. Finally, patients with LSM ≥11.5 kPa using the M probe or ≥11.0 kPa using XL probe were defined as having cirrhosis. Cutoff values were previously defined (71, 72). Ultrasound—guided liver biopsies were done by an experienced gastroenterologist.

The Clinical Hospital Rijeka Ethics committee approved this research under number 003-05/15-2/60. Fresh biopsy material was obtained by the Department of Pathology and Molecular Pathology, University Hospital Zurich and was approved by the local ethics committee (Kantonale Ethikkommission Zürich, KEK-ZH-Nr. 2013-0382 and BASEC-Nr. PB_2018-00252). We conducted the research in accordance and agreement with the international Conference on Harmonization guidelines on Good Clinical Practice and with the Declaration of Helsinki.

### Mice

Mice were strictly age- and sex-matched within experiments and were held in SPF conditions and handled in accordance with institutional, national and/or EU guidelines. Male mice (8–12 weeks old) were fed *ad libitum* with an NCD (SSNIFF) or an SSD enriched in fat (40% of calories derived from animal fat (Bregi), fructose (22% (SSNIFF)), and cholesterol (2% (SSNIFF)). Where indicated, mice were fed with either high fat diet (HFD) where 50% of calories were derived from pig fat (Bregi), high cholesterol diet (HCD) containing 2% of cholesterol (SSNIFF) or methionine and choline deficient diet (MCD) purchased from SSNIFF. All lines were kept as breeding colonies in the local animal facility in Rijeka, Croatia, under specific pathogen-free conditions. All animal experiments were done with approval from the University of Rijeka Medical Faculty Ethics Committee and Croatian Ministry of Agriculture, Veterinary and Food Safety Directorate, under number UP/I-322-01/21-01/31.

### Cell isolation

Mice were sacrificed by O_2_/CO_2_ intoxication followed by CO_2_ suffocation and perfused with PBS. Livers were collected and directly smashed through sieve or first cut into small pieces and digested with 1 mg/ml Collagenase IV (Sigma-Aldrich) and 2 mg/ml DNase in HBSS (with Ca^2+^/Mg^2+^) supplemented with 5% FBS (Sigma-Aldrich). The cell suspension was centrifuged (500g for 5 min). The leukocytes in the pellets were isolated by gradient centrifugation with 40 and 80% Percoll. Cells were collected, washed with RPMI, and centrifuged. Pellet was incubated for 3 min in ACK (Ammonium-Chloride-Potassium, Sigma Aldrich) buffer to lyse erythrocytes and resuspended in 3 % RPMI. Spleens were first passed through a 70 µm cell strainer, washed with 3 % RPMI and centrifuged. Blood was collected into an EDTA-containing tail vein, and PBMCs were isolated with Lymphoprep (Serumwerk Bernburg) gradient centrifugation. Hepatocytes used for subsequent FACS analysis were isolated by Collagenase I perfusion *in situ* through the portal vein and subsequent homogenization on magnetic stirrer. For the establishment of cultured primary hepatocytes, livers were processed by the step collagenase perfusion as previously described (73). In brief, mice were anesthetized with ketamine/xylazine (Vetoquinol). Upon cannulation, mice were perfused via inferior vena cava with pre-warmed perfusion buffer (HBSS (Sigma Aldrich) supplemented with 25 mM HEPES (Sigma Aldrich) and 0,5 mM EDTA (VWR Life Sciences)) until bleaching of the liver was achieved. Next, the perfusion buffer was omitted for digestion buffer (HBSS with 25 mM HEPES and 25 µg/mL Liberase™ Research Grade (Sigma-Aldrich)). In situ digestion was followed by the digestion in the Petri’s dish with Williams E medium (PAN Biotech). The liver sack was ruptured with forceps along the surface and cells were released using a cell scraper and then strained through a sieve. Separation of live primary hepatocytes was achieved by centrifugation in Percoll. Pellet was resuspended in Williams E medium and plated in 6-well plates. Primary murine hepatocytes were stimulated by either 200 µmol oleic acid (Sigma Aldrich), 200 µg/ml cholesterol (SSNIFF), 25 mM fructose (SSNIFF) and/or 25 µg/ml IL-17A (PreproTech) in 10% Williams E medium. Cells were treated for 48 hours and analyzed by quantitative PCR or confocal microscopy. Human peripheral blood mononuclear cells (PBMCs) were isolated from peripheral blood by density gradient centrifugation using Histopaque 1077 Density Gradient Medium (Sigma-Aldrich, St. Louis, USA).

### Flow cytometry

Cells were stained and analyzed in PBS containing 1% BSA and NaN_3_ and pretreated with Fc block (clone 2.4G2, produced in-house at the University of Rijeka). Fixable Viability Dye eFluor 780 (eBioscience) was used to exclude dead cells. For intracellular staining, cells were stimulated for 4 h *in vitro* with PMA and ionomycin (Sigma-Aldrich) supplemented with Brefeldin A (eBioscience), or GolgiPlug and GolgiStop (both 1:1000; BD Biosciences). For specific stimulation of γδ T cells through receptors, 96-well microtiter plates were pre-coated with αNKG2D (MI-6) and 145-2C11 hybridoma supernatant containing αCD3 was added in the stimulation mixture containing Brefeldin A and αCD3-PE-eFluor 610. Permeabilization and fixation of cells was done with the Fix/Perm kit (BD Biosciences). Bodipy^493/503^ (BD) staining was used according to manufacturer’s protocol to assess relative lipid content in cells. Staining of nuclear proteins (T-bet, Eomes, ROR-γt) was done with the FoxP3 staining buffer set (eBioscience). BrdU labeling and staining were performed according to the BrdU flow kit instructions (BD Biosciences). Most flow cytometry experiments were done on a FACSverse or FACSaria (BD Biosciences), or MACS Quant Analyzer 16 (Mitenyi Biotec). Human liver leukocytes were measured on 5L Cytek Aurora (Cytek). Dead cells and doublets were excluded from the analysis using SSC-A/H, FSC-A/H and a Fixable Viability Kit (LIVE/DEAD Blue, Thermo Fisher). FCS files were analyzed using FlowJo (TriStar) software.

### Histology

For immunohistology, tissues were fixed in 4% formalin for at least 48 h, dehydrated and paraffin embedded. Sections (2 µm) were cut, deparaffinized and antigen retrieval was performed by using 1 M sodium citrate buffer or a Tris-EDTA (pH=9) buffer. Sections were blocked with goat serum and then incubated with primary antibodies overnight at 4 ºC. After endogenous peroxidase block using 0,3% H_2_O_2_, slides were incubated for 1h with secondary antibody. Staining was visualized with DAB (Dako) and brief hematoxylin counterstaining. Collagen deposition was detected with Sirius red staining on deparaffinized slides stained with 0.1% Picro-Sirius Red solution (Sigma-Aldrich, USA) for 1h. Slides were washed in acidified water, dehydrated and mounted with Entellan (Sigma-Aldrich, USA). Basic structural parameters were observed on slides stained with hematoxylin and eosin. Stainings were quantified using ImageJ software (NIH) or by scoring of blinded samples by an expert pathologist according to established definitions (74). Immunopathology was quantified by adding the scores of infiltration foci, cysts and granulomas per vision field (200x magnification). For immunofluorescence, liver slides were deparaffinized and antigen retrieval was performed using Tris-EDTA buffer. Sections were blocked with 3% BSA and primary antibodies (Ki67 (Invitrogen) and CD45 (Cell Signaling)) were incubated overnight. Secondary antibodies were incubated for 1h at RT, nuclei were counterstained with 5’ DAPI incubation and slides were mounted with Mowiol 4-88. For confocal microscopy, primary murine hepatocytes were fixed in 4% PFA for 15 min at RT. Cells were washed twice with PBS and permeabilized for 5min using 0.1% TritonX. Cells were washed two times with PBS and incubated with 1.25 µg/ml Bodipy^493/503^ (BD Bioscience) for 30 minutes. Cells were washed twice with PBS and analyzed at room temperature with a Leica TCS SP8 confocal laser scanning microscope using an HC PL APO 40x/1.30 OIL CS2 objective and LasX acquisition software (version 3.5.6.21594) without gamma adjustments.

### Quantitative PCR

RNA was isolated from primary murine hepatocytes using NucleoZOL (MACHEREY-NAGEL) according to manufacturer’s protocol and cDNA was generated with a Reverse Transcription Core Kit (Eurogentec). The expression of mRNA was examined by quantitative PCR with a 7500 Fast Real Time PCR machine (ABI). The relative mRNA expression was normalized by quantification of HPRT housekeeping gene in each sample.

### RNAseq

Total RNA of liver samples was extracted using the Qiagen RNeasy Micro Kit according to the manufacturers’ protocol. Sequencing of 100 bp single reads was done on Illumina Novaseq 6000 (Illumina Inc., California, USA) at the Functional Genomics Center Zurich (FGCZ). The library was prepared with the Illumina Truseq Total RNA protocol. The resulting raw reads were evaluated for their quality by using FastQC and subsequently mapped to the mouse genome build GRCm39 using STAR aligner. FeatureCounts was used to quantify the read counts per gene based on GENCODE gene annotation version M26. Differences in gene expression levels between sample groups of interest were calculated as log2 fold changes using DESeq2 and genes exhibiting a false discovery rate (FDR)-adjusted *P* value<0.05 and absolute fold changes (FC) > 1.5 were considered significant. Hypergeometric over-representation analysis (ORA) based on Gene Ontology was applied on log-transformed and normalized counts using the clusterProfiler package. RNA sequencing data was deposited at the GEO database of NCBI under identifier GSE200482.

### Elisa

Serum samples were obtained from NCD and SSD-fed mice and concentrations of HMGB1 protein were determined by commercial HMGB1 ELISA colorimetric kit (Novus Biologicals) according to the manufacturer’s protocol. Plates were analyzed using a Mithras LB940 ELISA plate reader (Berthold technologies).

### In vivo experiments

VATectomy was done as previously described (28). Briefly, mice either underwent sham operation or had peri-epididymal fat pad removed (VATectomy). After two weeks mice were placed on an NCD or an SSD diet for 16 weeks. For adoptive transfers, WT (CD45.2) mice were NCD- or SSD-fed. After 14 days animals were injected i.v. with 5x10^5^ hepatic lymphocytes from NCD (CD45.1) and SSD (CD45.1/2) co-fed animals, mixed in a 1:1 ratio. 24 h after transfer, the ratio of donor cells in liver and spleen were determined by flow cytometry. For homing experiments, WT (CD45.1) mice were NCD- or SSD-fed for 2 weeks and injected i.v. with 5x10^5^ hepatic leukocytes from WT (CD45.1/2) and *Klrk1*^-/-^ (CD45.2) SSD co-fed animals, mixed in a 1:1 ratio. After 24 h, ratio between WT and *Klrk1*^-/-^ CD3^+^TCRδ^+^ and CD3^+^TCRδ^-^ cells was determined in spleen and liver by flow cytometry. For NK1.1 depletion experiment, mice were weekly treated with i.p. injection of InVivoMAb anti-mouse NK1.1 (clone PK136). After 16 weeks of NCD and SSD-feeding, PBS-injected control mice and anti-mouse NK1.1 treated mice were sacrificed and fibrosis/steatosis was determined as previously described. Labelling of circulating γδ T cells was performed using the i.v. injection of biotin-labeled anti-mouse CD45 antibody (clone 30-F11) (2 µg per mice). Mice were sacrified 5 minutes after i.v. injection, livers were perfused and analyzed using flow cytometry as previously described.

### Lipidomics

Lipidomic analysis was performed in collaboration with EMBL Metabolomics Core Facility (Heidelberg, Germany). Lipid and fatty acid extraction from the blood and liver was performed according to the protocol (PMID: 24820162). LC-MS/MS analysis was performed on a Vanquish UHPLC system coupled to an Orbitrap Exploris 240 high-resolution mass spectrometer (Thermo Scientific, MA, USA) in negative and positive ESI (electrospray ionization) mode.

### Statistics

Figures represent means and s.e.m. (depicted by error bars). To analyze statistical significance, we used either Student’s t-test, Mann-Whitney’s U test, one-way ANOVA, with Bonferroni’s post-test correction for multiple comparisons or simple linear regression. P values of <0.05 (*p<0.05, **p<0.01, and ***p< 0.001) were considered statistically significant.

## Supplementary Material

Supplementary Material

## Figures and Tables

**Table:** List of materials and reagents

Reagent or resource	Supplier/source	Product identification nr.
Armenian hamster anti-mouse monoclonal antibody TCR gamma/delta (GL3)	eBioscience	17-5711-82
Armenian hamster anti-mouse monoclonal antiobody TCR beta (H57-597)	eBioscience	12-5961-82
Rat anti-mouse CD4 (RM4-5)	eBioscience	63-0042-82
Armenian hamster anti-mouse CD3e monoclonal antibody (Clone 145-2C11)	eBioscience	12-0031-82
Armenian hamster anti-mouse CD69 monoclonal antibody (Clone H1.2F3)	eBioscience	11-0691-81
Armenian hamster anti-mouse gdTCR monoclonal antibody (Clone GL-3)	eBioscience	17-5711-82
Fixable Viability Dye eFluor™ 506	eBioscience	65-0865-14
Rat anti-mouse CD8a monoclonal antibody (53-6.7)	eBioscience	48-0081-82
Rat anti-mouse CD19 monoclonal antibody (1D3)	eBioscience	78-0193-82
Rat anti-mouse IL-17A monoclonal antibody (eBio17B7)	eBioscience	25-7177-82
Mouse anti-mouse CD45.2 monoclonal antibody (Clone 104)	eBioscience	47-0454-82
Mouse anti-mouse IFNγ monoclonal antibody (Clone AN-18)	eBioscience	14-7313-81
Rat anti-mouse IFNg monoclonal antibody (XMG1.2)	eBioscience	12-7311-82
Rat anti-mouse TNF monoclonal antibody (MP6-XT22)	eBioscience	53-7321-82
Rat anti-mouse CD49b monoclonal antibody (DX5)	eBioscience	13-5971-82
Armenian hamster anti-mouse CD27 monoclonal antibody (LG.7F9)	eBioscience	12-0271-82
Rat anti-mouse CD11b monoclonal antibody (M1/70)	eBioscience	25-0112-82
Mouse anti-mouse NK1.1 monoclonal antibody (PK136)	eBioscience	47-5941-82
Rat anti-mouse CD335 (Nkp46) monoclonal antibody (29A1.4)	eBioscience	11-3351-82
Rat anti-mouse CD68 monoclonal antibody (FA-11)	eBioscience	25-0681-82
Rat anti-mouse CD68 monoclonal antibody (FA-11)	eBioscience	25-0681-82
Armenian hamster anti-mouse CD11c monoclonal antibody (N418)	eBioscience	63-0114-82
Ly-6G	eBioscience	48-9668-82
Rat anti-mouse Siglec F monoclonal antibody (1RNM44N)	eBioscience	46-1702-82
Rat anti-mouse Ly6G monoclonal antibody (1A8-Ly6g)	eBioscience	48-9668-82
Rat anti-mouse Ly6C monoclonal antibody (HK1.6)	eBioscience	12-5932-82
Rat anti-mouse MHC II monoclonal antibody (M5/114.15.2)	eBioscience	11-5321-85
Rat anti-mouse F4/80 (BM8)	eBioscience	17-4801-82
CD314 (NKG2D) (CX5)	eBioscience	25-5882-82
Rat anti-mouse/human/rhesus monkey ROR gamma (t) monoclonal antibody (AFKJS-9)	eBioscience	**61-6988-82**
Mouse anti mouse/human/rhesus monkey T-bet monoclonal antibody (4B10)	eBioscience	12-5825-82
Rat anti-mouse/human Eomes monoclonal antibody (Dan11mag)	eBioscience	53-4875-82
Armenian hamster anti-mouse TCRVγ1 monoclonal antibody (2.11)	Biolegend	141105
Armenian hamster anti-mouse TCRVγ2 (UC3-10A6)	Biolegend	137703
Sirian hamster anti-mouse TCRVγ3 (536)	Biolegend	137503
Mouse anti-mouse TCRVγ4 monoclonal antibody (UC3-10A6)	Biolegend	160602
Rat anti-mouse CD122 monoclonal antibody (TM-b1)	eBioscience	11-1222-82
Rat anti-mouse CD44 monoclonal antibody (IM7)	eBioscience	47-0441-82
Mouse anti- mouse/human/mammal/rat CD25 monoclonal antibody (CD25-3G10)	eBioscience	MA1-35144
Armenian hamster anti-mouse CD69 (H1.2F3)	eBioscience	25-0691-82
Rat atni-mouse CD127 monoclonal antibody (A7R34)	eBioscience	12-1271-82
Rat anti-mouse CD16/32 monoclonal antibody (93)	eBioscience	16-0161-82
Mouse anti-mouse CD45.2 monoclonal antibody (104)	eBioscience	56-0454-82
Rat anti-mouse IgM (II/41)	eBioscience	12-5790-82
Rat anti-dog/monkey/human/mouse Ki-67 monoclonal antibody (SolA15)	eBioscience	11-5698-82
Rat anti-mouse CD206 monoclonal antibody (MR5D3)	BD Biosciences	MA5-16870
Mouse anti-human gdTCR monoclonal antibody (B1.1)	eBioscience	TCR2720
Mouse anti-human CD161 (HP-3G10)	eBioscience	12-1619-42
Rat anti-mouse ROR gamma t monoclonal antibody (B2D)	eBioscience	12-6981-82
Mouse anti-human NKG2D monoclonal antibody (1D11)	eBioscience	46-5878-42
Mouse anti-human CD45 monoclonal antibody (HI30)	eBioscience	MHCD4531
Mouse anti-human IL-17A monoclonal antibody (eBio64DEC17)	eBioscience	17-7179-42
Mouse anti-human CD8 monoclonal antibody (OKT8)	eBioscience	47-0086-42
Mouse anti-human CD4 monoclonal antibody (OKT4)	eBioscience	**63-0048-42**
Mouse anti-human CD3 monoclonal antibody (OKT3)	eBioscience	67-0037-42
Vα7.2 (REA179)	Miltenyi	130-100-188
Rat anti-mouse IFNγ (biotinylated)monoclonal antibody (Clone R4-6A2)	eBioscience	13-7312-81
Rat anti-mouse IFNβ monoclonal antibody (Clone 7F-D3)	abcam	ab24324
Rat anti-mouse TNFα monoclonal antibody (Clone MP6-XT22)	eBioscience	11-7321-82
Rat anti-mouse CD11b monoclonal antibody (M1/70)	BD	612800
Rat anti-mouse CD11b monoclonal antibody (M1/70)	Biolegend	101239
Armenian hamster anti-mouse CD11cmonoclonal antibody (N418)	eBioscience	35-0114-82
Rat anti-mouse CD127 monoclonal antibody (A7R34)	Biolegend	135027
Rat anti-mouse CD16/32 monoclonal antibody (93)	Biolegend	101301
Rat anti-mouse CD172a monoclonal antibody (P84)	BD	740282
Rat anti-mouse CD19 monoclonal antibody (1D3)	BD	612971
Rat anti-mouse CD206 monoclonal antibody (C068C2)	Biolegend	141734
Armenian hamster anti-mouse CD27monoclonal antibody (LG.3A10)	Biolegend	124216
Rat anti-mouse CD3 monoclonal antibody (17A2)	Biolegend	100220
Rat anti-mouse CD4 monoclonal antibody (GK1.5)	Biolegend	100473
Rat anti-mouse CD44 monoclonal antibody (IM7)	BD	612799
Rat anti-mouse CD45 monoclonal antibody (30-F11)	Biolegend	103216
Rat anti-mouse CD45 monoclonal antibody (30-F11)	BD	564279
Hamster anti-mouse CD49a monoclonal antibody (Ha31/8)	BD	740144
Rat anti-mouse CD49b monoclonal antibody (DX5)	BioLegend	108918
Rat anti-mouse CD62L monoclonal antibody (MEL-14)	BioLegend	104433
Mouse anti-mouse CD64 monoclonal antibody (X54-5/7.1)	BioLegend	139309
Armenian hamster anti-mouse CD69monoclonal antibody (H1.2F3)	BioLegend	104530
Rat anti-mouse CD8 monoclonal antibody (53-6.7)	BD	612898
Rat anti-mouse CD88 monoclonal antibody (20/70)	BD	747227
Rat anti-mouse CD90 monoclonal antibody (30-H12)	BioLegend	105329
Rat anti-mouse CD90 monoclonal antibody (30-H12)	BioLegend	105327
Mouse anti-mouse CX3CR1 monoclonal antibody (SA011F11)	BioLegend	149027
Rat anti-mouse ESAM monoclonal antibody (1G8/ESAM)	BioLegend	136203
Rat anti-mouse F4/80 monoclonal antibody (BM8)	BioLegend	123135
Rat anti dog/cat/pig/mouse Foxp3 monoclonal antibody (FJK-16s)	eBioscience	61-5773-82
Rat anti-mouse Ly6C monoclonal antibody (HK1.4)	BioLegend	128037
Rat anti-mouse Ly6G monoclonal antibody (1A8)	BD	565707
Rat anti-mouse/human MHCII monoclonal antibody (M5/114.15.2)	BioLegend	107616
Rat anti-mouse/human MHCII monoclonal antibody (M5/114.15.2)	BioLegend	107622
Mouse anti-mouse NK1.1 monoclonal antibody (PK136)	Biolegend	108724
Mouse anti-mouse NK1.1(monoclonal antibody PK136)	BioLegend	108745
Rat anti-mouse Siglec-F monoclonal antibody (E50-2440)	BD	562757
Rat anti-mouse Siglec-F monoclonal antibody (E50-2440)	Biolegend	747316
anti-mouse Siglec-H monoclonal ibody (551)	oscience	46-0333-80
Hamster anti-mouse TCRb monoclonal antibody (H57-597)	Biolegend	109209
Rat anti-mouse Tim4 monoclonal antibody (RMT4-54)	Biolegend	130007
Mouse anti-mouse XCR1 monoclonal antibody (ZET)	Biolegend	148220
Rat anti-mouse CD192 (CCR2)-PE	BD Pharmingen	747963
Rat anti-mouse CD182 (CXCR2)-BV421	BD Pharmingen	566622
Recombinant human anti-mouse IL-1β pro-form antibody (REA577)	Miltenyi Biotec	130-109-043
Recombinant human anti-mouse IL-6 antibody (REA1034)	Miltenyi Biotec	130-117-582
Rat anti-mouse CD45 monoclonal antibody (30-F11)	oscience	13-0451-82
Mouse anti-human CD16 monoclonal antibody (3G8)	BD Pharmingen	612944
Mouse anti-human CD161 monoclonal antibody (HP-3G10)	BD Pharmingen	749223
Mouse anti-human CD19 monoclonal antibody (HIB19)	eBioscience	62-0199-42
Mouse anti-human CD45 monoclonal antibody (2D1)	BioLegend	368549
Mouse anti-human CD3 monoclonal antibody (UCHT1)	BD Pharmingen	612895
Mouse anti-human CD8a monoclonal antibody (RPA-T8)	BioLegend	301038
Mouse anti-human CD4 monoclonal antibody (RPA-T4)	BioLegend	300519
Mouse anti-human CD56 monoclonal antibody (5.1H11)	BioLegend	362543
Mouse anti-human TCR Vα7.2 monoclonal antibody (3C10)	BioLegend	351705
Mouse anti-human IL-17A monoclonal antibody (BL168)	BioLegend	512320
Fc block (clone 2.4G2)	Produced in house	568093
Rat anti-mouse NKG2D (MI-6)	eBioscience	16-5880-82
goat anti-rat IgG-HRP	Jackson ImmunoResearch	112-035-003
donkey anti-goat IgG-HRP	Invitrogen	A15999
αSMA 1A4	Invitrogen	**14-9760-82**
αCD3 145-2C11	eBioscience	16-0032-82
Ki-67 (TEC-3)	Dako	M7240
Goat anti-human IL-17A	R&D	AF-317-NA
αMICA/B (BAMOI)	Made in house (AS)	
αULBP1 (AUMO2)	Made in house (AS)	
αULBP3 (CUMO3)	Made in house (AS)	
goat anti-mouse IgG-HRP	Jackson ImmunoResearch	115-035-003
Rabbit anti-mouse CD45 (D3F8Q)	Cell Signaling	70257S
Goat anti-mouse IgG Alexa Fluor-647	Abcam	ab150083
Rat anti-mouse Ki-67	Invitrogen	14-5698-82
Goat anti-rat IgG Alexa Fluor 594	Invitrogen	1008647
Capture antibody IL-17A	eBioscience	eBio17CK15A5
biotinylated detection antibody	eBioscience	eBio17B7
Polyvinyl alcohol 4-88	Fluka Chemika	81381
DAPI (4’,6-Diamino-2-Phenylindole, Dilactate)	BioLegend	422801
mouse tetramer MR1 5-OP-RU-PE	NIH Tetramer Facility	
mNKG2D-hFc	produced in house	
Brefeldin A	eBioscience	00-4506-51
TCRVγ6 (17D1) was made in house		00-4506-51
Citrate (tri-sodium citrate dehydrate)	Kemika	1405407
Collagenase I	Sigma Aldrich	1148089
Collagenase (IV) D	Sigma Aldrich	11088858001
DMEM (4.5 g/L glucose, w L-glutamine)	Pan Biotech	P04-01549
DMSO (Dimethyl sulphoxide)	Gram-mol	P04-03550
DPBS (Dulbecco’s phosphate-buffered saline)	Pan Biotech	P120601
Fetal Bovine Serum (pretested for ES cells)	Pan Biotech	P04-36500
Fetal Bovine Serum (FBS Standard)	Pan Biotech	2602-P260328P30-2602
Fixation/Permeabilization Concentrate	Invitrogen	P30-3306
Fixation/Permeabilization Diluent	Invitrogen	00-5123
Formaldehyde (4% NB)	Biognost	00-5223
DNase		FNB4
HBSS	Sigma Aldrich	H8264
HBSS w/o	Sigma Aldrich	H55037C
IC Fixation Buffer	Invitrogen	P04-80100
ACK (Ammonium-Chloride-Potassium)	Sigma Aldrich	254134, 237205
Lymphoprep	Serumwerk Bernburg	04-03-9391/01
Ionomycin	Sigma Aldrich	407952
HEPES	Merck	7365-45-9
Mayer’s hematoxylin	Sigma Aldrich	MDL# MFCD00078111
Streptavidin-PE-Cyanine5	eBioscience	15-4317-82
Streptavidin- Super Bright 436	eBioscience	62-4317-82
Streptavidin-Super Bright 645	eBioscience	64-4317-82
Streptavidin-APC-eFluor780	eBioscience	47-4317-82
ketamine	Vetoquinol	Vm 08007/4090
xylazine	Vetoquinol	VTQ417633
NucleoZOL	Macherey-Nagel	593-84-0
o-Phenylenediamine dihydrochloride(OPD)	Sigma Aldrich	740404
Penicillin-Streptomycin	Pan Biotech	P4664
Percoll	Cytiva	P06-07100
Permeabilization Buffer (10x)	Invitrogen	17089101
EDTA VWR Life Sciences	(VWR Life Sciences)	00-8333
PMA (Phorbol 12-myristate 13- acetate)	Sigma Aldrich	P1839
RBC Lysis Buffer	eBioscience	00-4333-57
Liberase™ Research Grade	Sigma-Aldrich	00430054
Williams E medium PAN Biotech	PAN Biotech	P04-29500
Oleic acid	Merck	05508
RPMI 1640	Pan Biotech	P04-18047
Sirius Red (Direct Red 80)	Sigma Aldrich	P04-22100
Cholesterol	SSNIFF	R143C040
Sodium Pyruvate	Sigma Aldrich	P2256
Sunflower Seed Oil	Sigma Aldrich	47123
Topro3	Invitrogen	S5007
Trypsine/EDTA (10x)	Pan Biotech	T3605
Tween20	Sigma Aldrich	P10-024100
IL-17A	PreproTech	P1379
Fructose	SSNIFF	R111F040
Fructose	SpecialIngredients	5056191008429
MCD (methionine and choline deficient diet)	SSNIFF	E15653-94
Albumin fraction V	Roth	T844.3
NaN_3_	Alfa Aesar	U17D012
Fixable Viability Dye eFluor 780	eBioscience	65-0865-14
Fixable Viability Dye eFluor 506	eBioscience	65-0866-14
Bodipy^493/503^	BD Biosciences	D3922
Fixable Viability Kit (LIVE/DEAD Blue)	Thermo fisher	L23105
EDTA	Kemika	1136808
Tris buffer	Roth	4855.2
goat serum	Dako	X090710-8
H_2_O_2_	Gram mol	P154903
DAB	Dako	K3468
Entellan	Sigma-Aldrich, USA	1079600500
Instant Eosin	Thermo Scientific	6765540
TritonX	Sigma Aldrich	X100
SYBR Green dye	Eurogentec	UF-NSMT-B0701
Glacial acetic acid	Kemika	1500301
Picric acid	Sigma Aldrich	319287
Reverse Transcription Core Kit	Eurogentec	RT-RTCK-03
Takyon No Rox SYBR MasterMix dTTP Blue	Eurogentec	43-695
BrdU flow kit	BD Biosciences	559619
FoxP3 staining buffer set	eBioscience	00-5523-00
Fix/Perm kit	BD Biosciences	554714
Balbc	Jackson Laboratories	JAX:000651
C57BL/6 (B6; line 664)	Jackson Laboratories	JAX: 000664
C57BL/6J CD45.1 (2014)	Jackson Laboratories	JAX: 002014
*LysM*Cre (4781)	Jackson Laboratories	JAX: 004781
*Alb*Cre (3574)	Jackson Laboratories	JAX: 003572
GFAP-Cre line 77.6 (024098)	Jackson Laboratories	JAX: 024098
*Cα*^*fl*^/^*fl*^	Produced in house	PMID: 11447257
TCRd^-/-^	Prof. AdrianHayday; London, England	JAX: 002116
*Klrkl*-/-	Produced in house	PMID: 19631564
*Klrkl*fl/^fl^	Produced in house	PMID: 21898152
*lll7ra*fl/^fl^	Ari Waisman;Mainz, Germany	PMID: 22951726
Del^Cre^	Taconic	JAX: 0012524
Mr1–/– *Rorcgt-GFPTG *B6-MAITCAST	Olivier Lantz; Paris,France	PMID: 6524590
Mr1+/+ *Rorcgt-GFPTG *B6-MAITCAST	Olivier Lantz; Paris,France	PMID: 6524590
*NCR1Cre*	Veronica Sexl;Vienna, Austria	PMID: 21127177
*Cd1d-/-*	Thomas Wunderlich, Max Planck, Germany	JAX: 008881
*Cd4*Cre	D. Littman; New York, NY, USA	PMID: 12121668
*Hcst*-/-	M. Colonna; St.Louis, MO, USA	PMID: 19247984
HPRT forward 5’- TGAAGAGCTACTGTAATGATCAGTCAAC-3’	Metabion International AG	Custom
HPRT reverse 5’-AGCAAGCTTGCAACCTTAACCA-3’)	Metabion International AG	Custom
(PanRae1ε forward5’-CAGGTGACCCAGGGAAGATG-3’;	Metabion International AG	Custom
PanRae1ε reverse 5’-CTCAACTCCTGGCACAAATCG-3’):,	Metabion International AG	Custom
MULT1 (MULT1 forward 5’-CTCATAGGAACAGCATGA-3’	Metabion International AG	Custom
MULT1 reverse 5’-TCCTGTGAAATGTTTGTC-3’),	Metabion International AG	Custom
H60 (H60 forward 5’-GAGCCACCAGCAAGAGCAA-3’	Metabion International AG	Custom
H60 reverse 5’-CCAGTATGGTCCCCAGATAGCT-3’),	Metabion International AG	Custom
CXCL2 (CXCL2 forward 5’- CCCAGACAGAAGTCATAGCCAC-3’	Metabion International AG	Custom
CXCL2 reverse 5’- CTTCCGTTGAGGGACAGCAG-3’),	Metabion International AG	Custom
CXCL1 (CXCL1 forward 5’- CAGACCATGGCTGGGATTCA-3’;	Metabion International AG	Custom
CXCL1 reverse 5’-ACTTGGGGACACCTTTTAGCAT-3’),	Metabion International AG	Custom
CCL2 (CCL2 forward 5’-GCCTGCTGTTCACAGTTGC-3’;	Metabion International AG	Custom
CCL2 reverse 5’- TTGAGCTTGGTGACAAAAACTAC-3’)	Metabion International AG	Custom
CXCL10 (CXCL10 forward 5’-ATGACGGGCCAGTGAGAATG-3’;	Metabion International AG	Custom
CXCL10 reverse 5’-AGGAGCCCTTTTAGACCTTTTT-3’)	Metabion International AG	Custom
7500 Software	Applied Biosystems; Life Technologies	
Fiji/ImageJ	*Nature Methods*,*9*(7), 676−682.	doi:10.1038/nmeth.2019
FlowJo	FlowJo LLC, BD Life Sciences	https://imagej.net/software/fiji/
GraphPad Prism	GraphPad Software	
MicroWin	Berthold Technologies	
Cell^ B Soft Imaging System	Olympus	
Mithras LB940 ELISA plate reader	Berthold technologies	

## Data Availability

All unique materials and reagents will be made available by the corresponding author upon reasonable request. RNA-sequencing datasets generated in this paper are available under accession code GSE200482.

## One Sentence Summary

Tissue resident γδ T cells mediate the transition from steatosis to steatohepatitis in metabolic-associated fatty liver disease.
