# Supplementary material for: NKG2D-mediated detection of metabolically stressed hepatocytes by innate-like T cells is essential for initiation of NASH and fibrosis
Source: Sci Immunol. Author manuscript; Available in PMC 2024 Feb 12. (PMC7615627; doi:10.1126/sciimmunol.add1599)
Supplement: Supplementary Material [file EMS193760-supplement-Supplementary_Materials.pdf]

Supplementary Figure 1

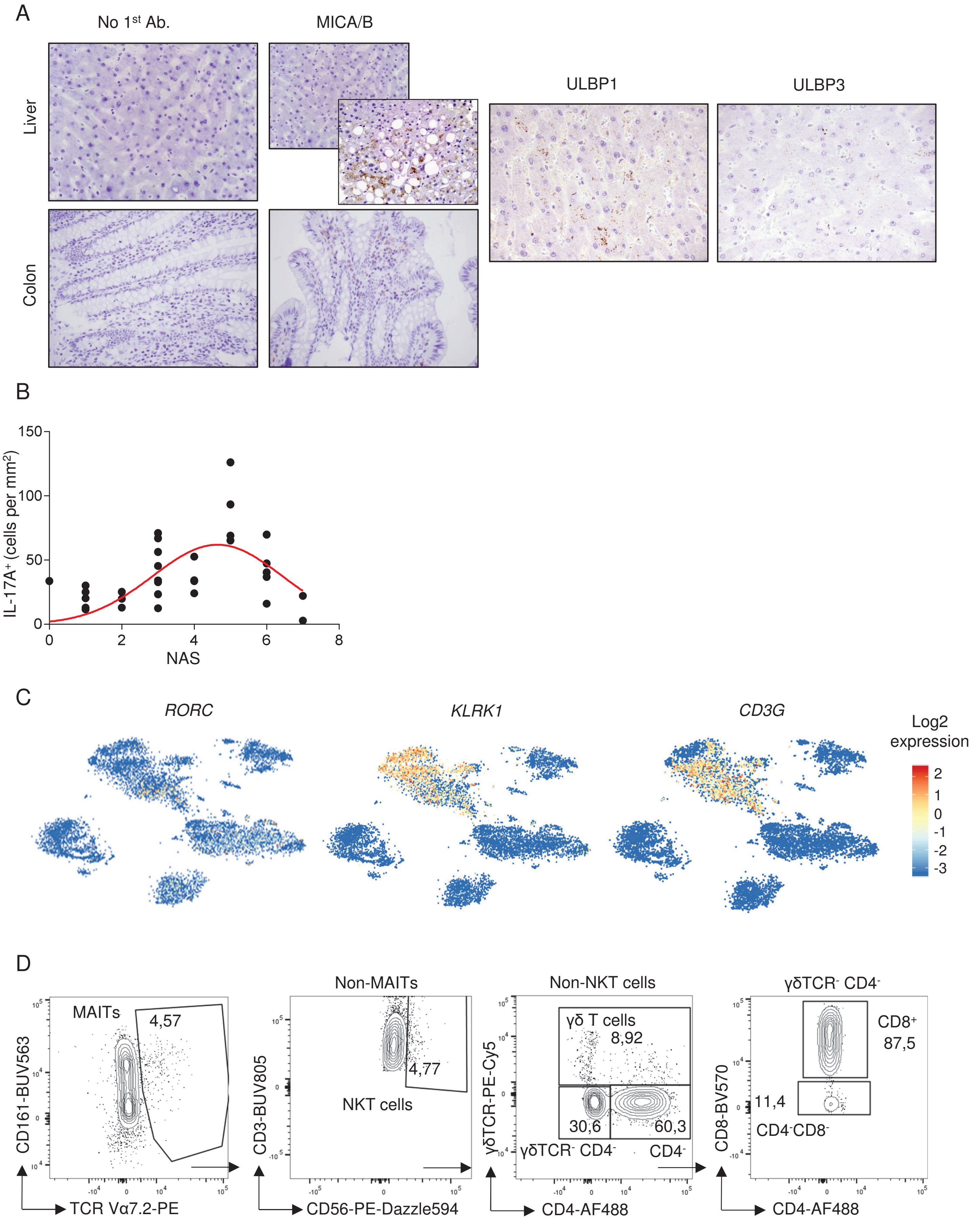

Figure S1. Human NASH is associated with the induction of IL-17A. (a) Human liver biopsy material of patients with MAFLD was stained for the NKG2D ligands MICA/B, or ULBP1 and ULBP3. In addition, healthy colon tissue, known to express MICA/B, was stained as a control. Samples stained with secondary antibody only were included as a negative control. Scale bars indicate 100 µm. (b) Human biopsy material of patients with MAFLD was stained for IL-17A. Shown is the correlation of the number of IL-17 producing cells with the level of steatosis, grade of NASH or the NAS-score (n=35). Red line shows non-linear regression using least squares fit. (c) Expression of indicated genes on human liver leukocytes obtained from a publicly available single cell RNA sequencing data of human liver biopsy material deposited in the Gene Expression Omnibus (GEO) with the accession code GSE124395. http://human-liver-cell-atlas.ie-freiburg.mpg.de/. (d) Gating strategy of data shown in 1e on human liver biopsy material.

Supplementary Figure 2

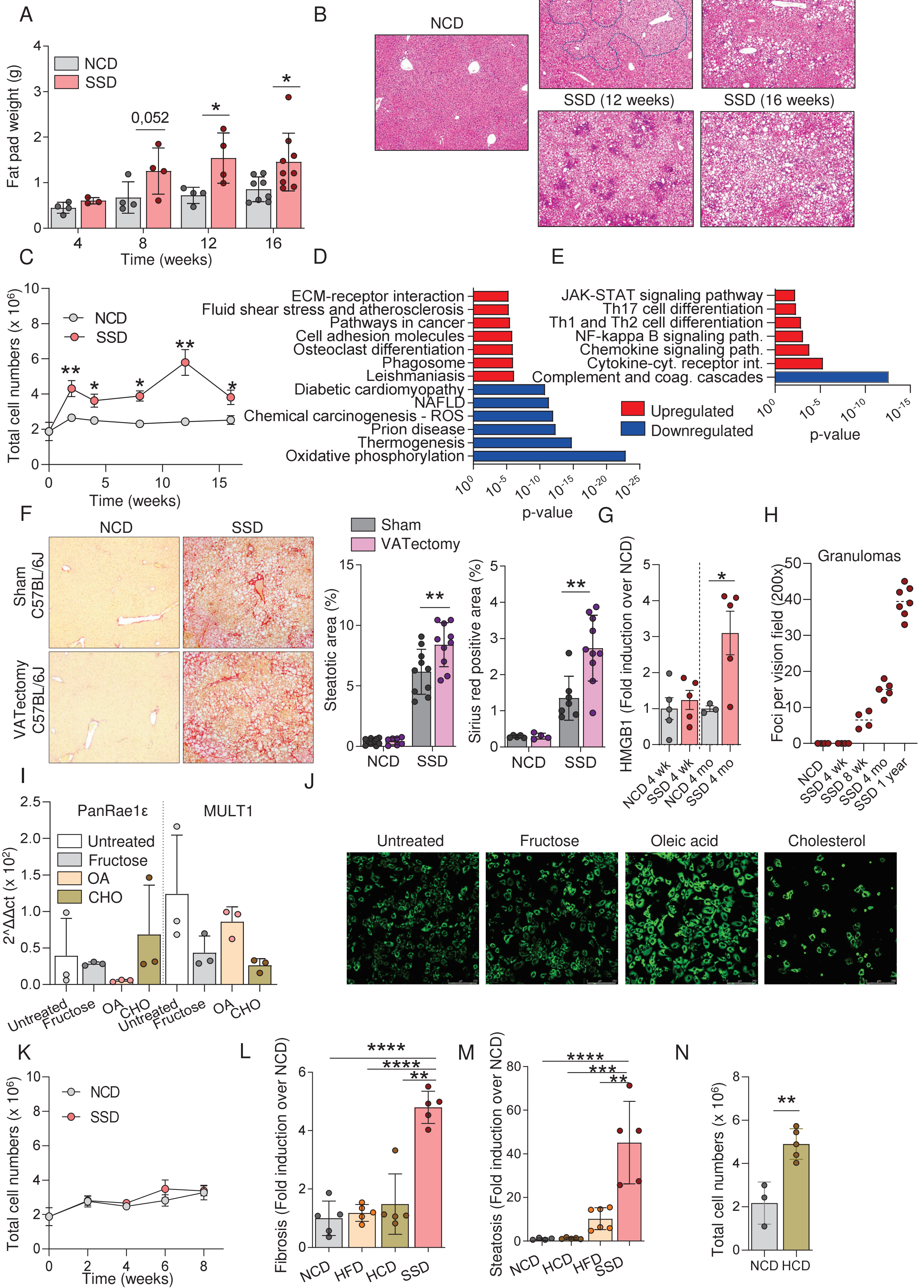

Figure S2. NKG2D ligand induction depends on cholesterol accumulation. (a-d) WT mice were fed a Steatosis and Steatohepatitis Diet (SSD) or a Normal Chow Diet (NCD) for 2 to 16 weeks. (a) quantification of abdominal obesity in mice by analysis of perigonadal fat pads (n=4-8). (b) Representative liver slides (200 X) of animals fed for 16 weeks with SSD, stained with H&E featuring hepatocyte distension (dashed line), steatosis (arrows), infiltration of inflammatory cells (arrowheads) and fibrosis (dots). (c) quantification of total liver leukocyte numbers over time (n=5). (d-e) Total liver lysates were analyzed by RNA sequencing after 3 weeks of NCD or SSD feeding (n=4-5). (d) KEGG-Gene pathways most affected by SSD feeding. (e) Inflammation-related KEGG-gene pathways affected by SSD feeding. (f) Mice were subjected to either VATectomy (removal of abdominal fat) or were sham operated. After recovery, they were fed an SSD or an NCD diet for 16 weeks. Representative liver slides stained with Sirius Red (200 X) and liver fibrosis quantification (n=4-10). (g) WT mice were fed an NCD or an SSD for the indicated periods and serum concentration of high mobility group box 1 (HMGB1) protein was measured by ELISA. Relative increase of concentration within SSD over NCD is shown. (h) WT mice were fed an SSD for the indicated periods. Granulomas were quantified using HE-stained histological liver slides. Graph shows the average number of granulomas observed in individual fields of view at 200x magnification. (i) Primary mouse hepatocytes were cultured for 48h in the presence of fructose, oleic acid or cholesterol. NKG2D ligand expression was quantified by qPCR and normalized to HPRT (n=3). (j) Representative images of cells stained with BODIPY and analyzed by confocal microscopy. (k) Leukocyte infiltration in livers of mice fed with NCD, or SSD lacking cholesterol was quantified over time. (l-m) Mice were fed with NCD, HFD or SSD. After 16 weeks, Sirius Red staining of liver slides was performed to quantify the levels of (l) liver fibrosis and (m) liver steatosis. (n) Total leukocyte cell count in mice fed an NCD or High Cholesterol (HCD) diet for 16 weeks. (b, f, j) Scale bars indicate 250 µm. The data are representative of two pooled experiments (c,f), or at least two independent experiments (a, g-n) and show means ±s.e.m. Statistical significance was determined by ANOVA with Bonferroni post-testing (l,m) and unpaired t-test. Statistical significance was defined as $^{*}p<0.05$; $^{**}p<0.01$; $^{***}p<0.001$.

Figure S3

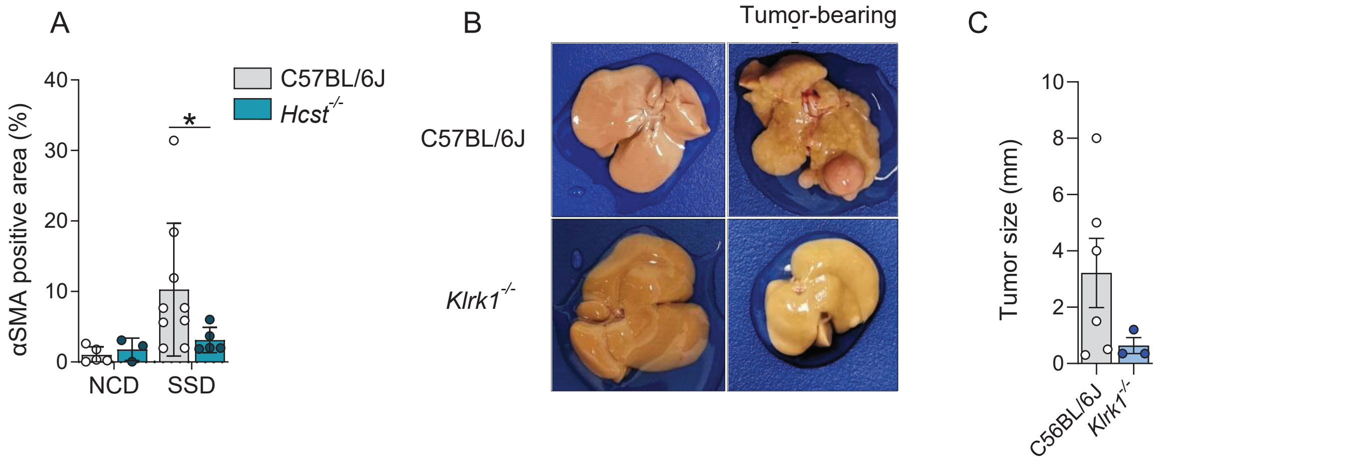

Figure S3. NKG2D engagement is essential for development of liver fibrosis in context of NASH. (a) WT and *Hcst*$^{-/-}$ (DAP10-deficient) were fed an NCD or an SSD diet for 16 weeks. Quantification of hepatic stellate cell activation by αSMA staining (n = 5-9). (b-c) WT and *Klrk1*$^{-/-}$ mice were fed an NCD or an SSD diet for 52 weeks and livers were analyzed. (b) Shown are livers from tumor-bearing mice and animals without visible malignancies. (c) Size of macroscopically observed tumors in livers. The data are representative of at least two independent experiments. Shown are means +/- s.e.m. Statistical significance was determined by unpaired t-test. Statistical significance was defined as *p<0.05; **p<0.01.

Supplementary Figure 4

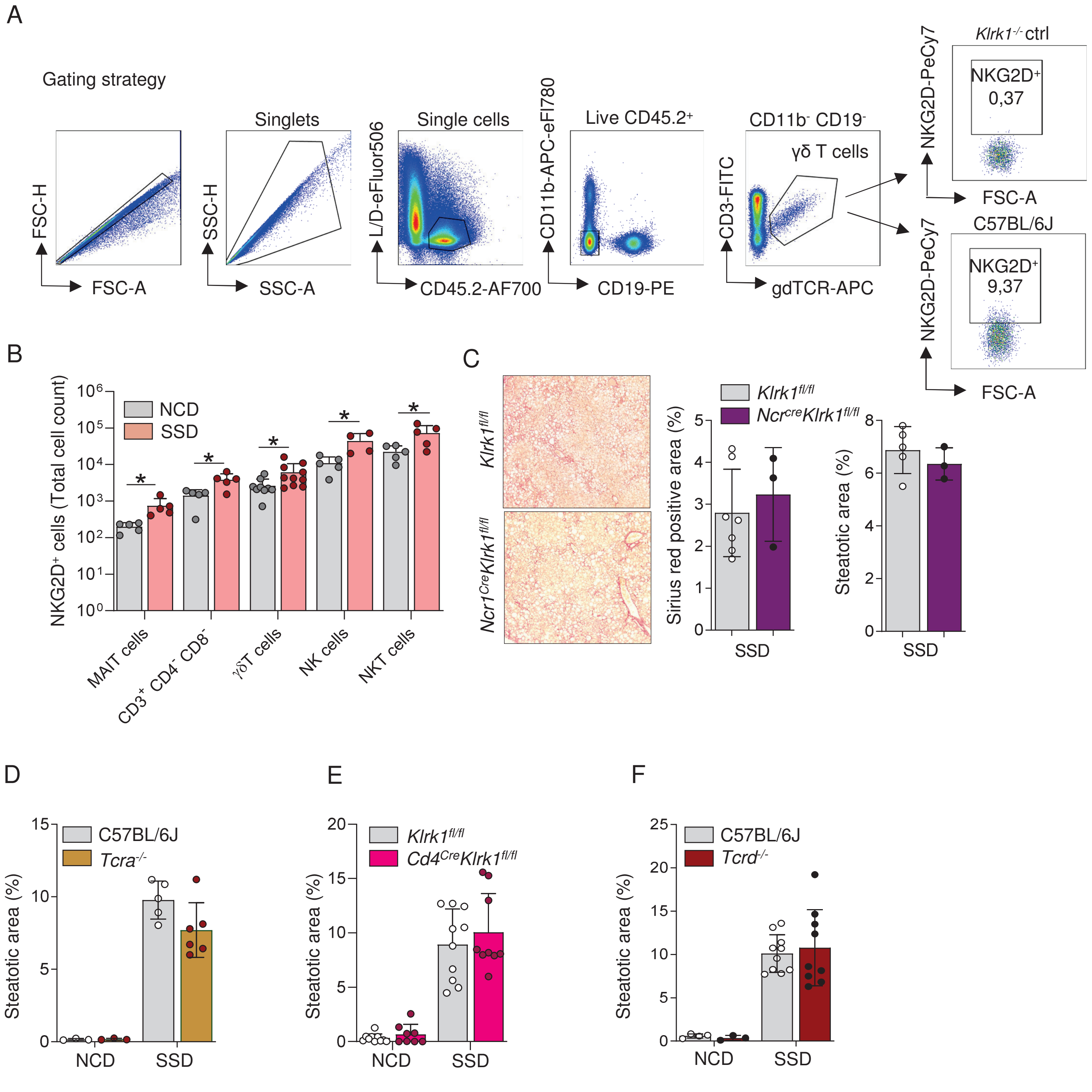

Figure S4. γδ T cells mediate fibrosis in diet-induced NASH. (a-b) WT mice were fed with an NCD or SSD for 18 days and NKG2D expression was quantified in key hepatic leukocyte populations by flow cytometry (n=5-10). (a) Gating strategy of data shown in 4a. (b) Absolute numbers of indicated NKG2D-expressing lymphocyte populations. (c) *Ncr1*^{Cre}*Klrk1*$^{Flox/Flox}$ and *Klrk1*$^{Flox/Flox}$ controls were fed an SSD diet for 16 weeks. Shown are representative liver slides stained with Sirius Red (200 X). Scale bars indicate 250 µm. Quantification of fibrosis and steatosis is shown(n = 3-7). (d) WT and *Tcra*$^{-/-}$ mice were fed an SSD diet for 16 weeks and livers were analyzed by histology. Quantification of steatosis is shown (n = 5-6). (e) *CD4*^{Cre}*Klrk1*$^{Flox/Flox}$ and *Klrk1*$^{Flox/Flox}$ controls were fed an SSD diet for 16 weeks and livers were analyzed by histology. Quantification of steatosis is shown (n=9-10). (f) WT and *Tcrd*$^{-/-}$ mice were fed an SSD diet for 16 weeks and livers were analyzed by histology. Quantification of steatosis is shown (n=9-10). The data are shown as two pooled experiments (e-f), or representative of at least two independent experiments (b-c, d). Shown are means +/- s.e.m. Statistical significance was determined by unpaired t-test. Statistical significance was defined as *p<0.05

Supplementary Figure 5

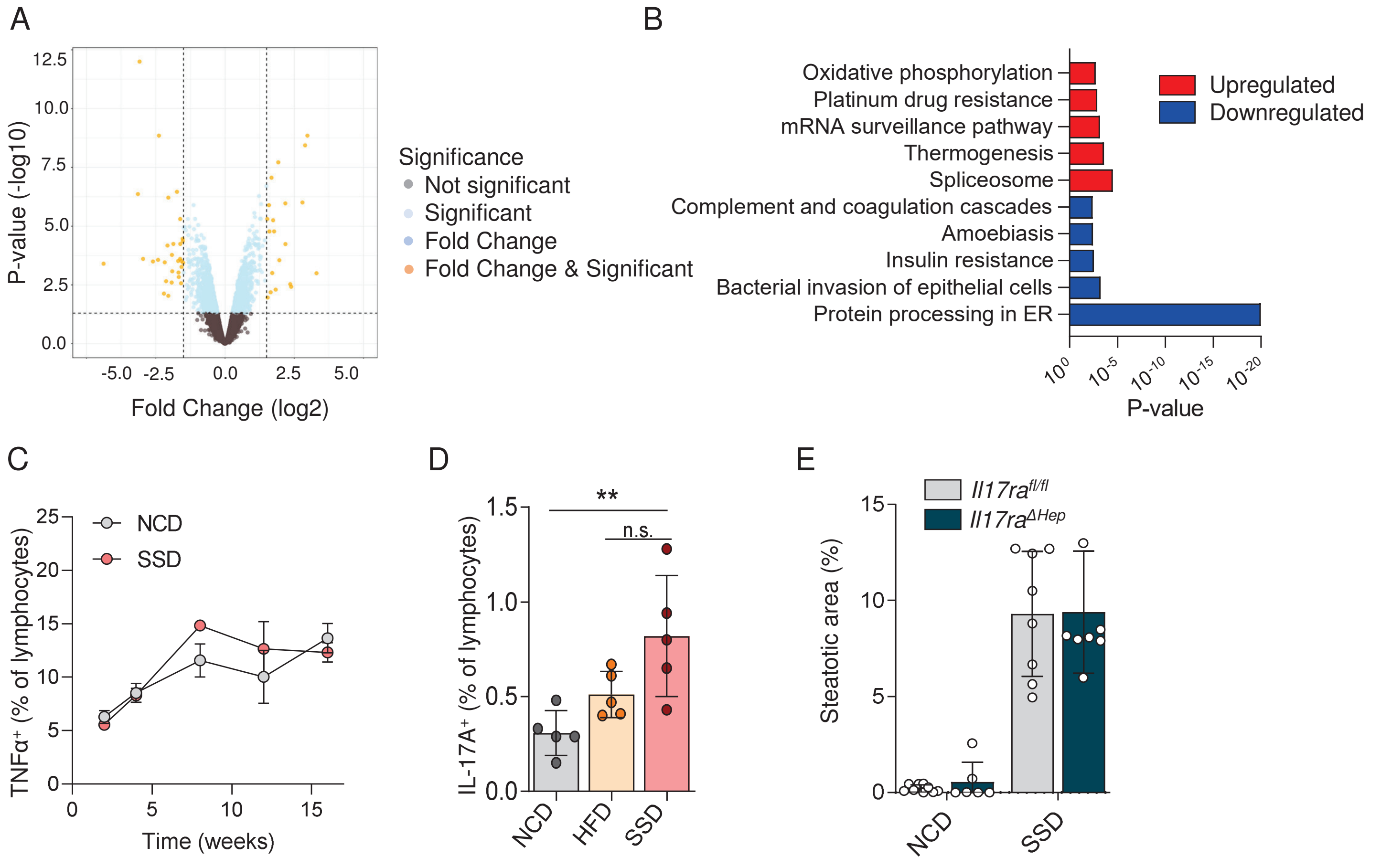

Figure S5. Diet-induced NASH, but not Diet-induced obesity is associated with an increase of IL-17A production. (a-b) Total liver lysates of NCD-fed WT and *Klrk1*$^{-/-}$ mice were analyzed by RNA sequencing. (a) Volcano plot of differentially expressed genes. (b) KAGG-gene pathways most affected by NKG2D-deficiency. (c) WT mice were fed with an NCD or SSD diet. At indicated time points, liver leukocytes were restimulated *in vitro* with PMA/Ionomycin and TNF production was measured by flow cytometry (n = 5). (d) WT mice were fed with a NCD, HFD or SSD diet. After 16 weeks, liver leukocytes were restimulated *in vitro* with PMA/Ionomycin and IL-17A production was measured by flow cytometry (n = 5). (e) *Il17ra*$^{fl/fl}$ and Albumin^{Cre}*Il17ra*$^{fl/fl}$ (*IL17ra*$^{\Delta Hep}$) mice were fed an NCD or an SSD diet for 16 weeks. Shown is quantification of liver steatosis of slides stained with Sirius Red (n = 7-8). The data are representative of at least two independent experiments (c-e), or one experiment using 3-5 biological replicates (a-b). Shown are means +/- s.e.m. Statistical significance was determined by unpaired t-test. Statistical significance was defined as *p<0.05; **p<0.01; ***p<0.001.

Supplementary Figure 6

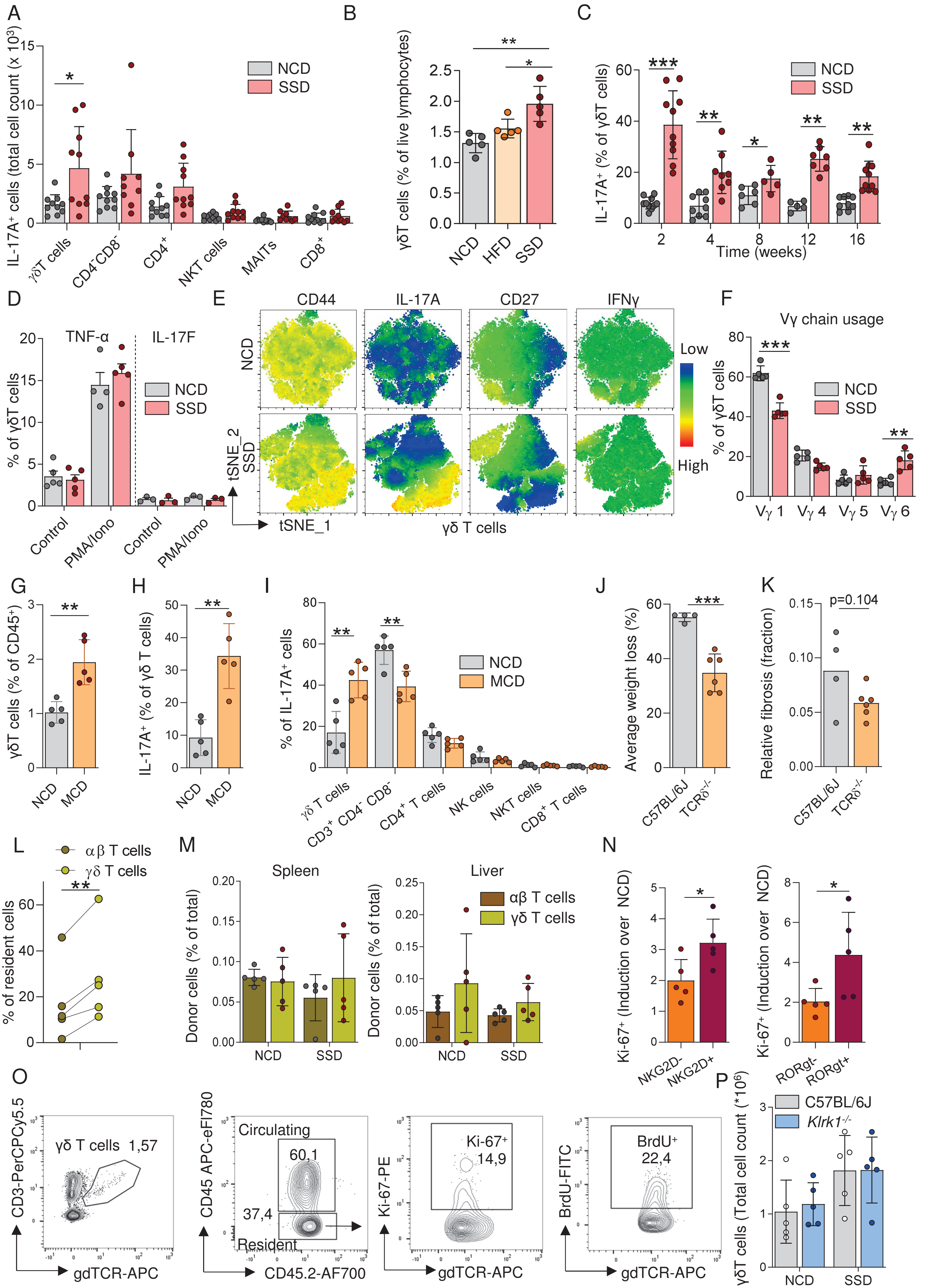

Figure S6. NKG2D stimulation promotes IL-17A production by hepatic γδ T cells in the context of NASH. (a) WT mice were fed with an NCD or SSD diet and after 18 days liver leukocytes were restimulated *in vitro* with PMA/Ionomycin. IL-17A production was analyzed by flow cytometry. Shown is the quantification of absolute numbers of IL-17A producing leukocytes in the liver (n=10). (b) WT mice were fed with a NCD, HFD or SSD diet. After 2 weeks, γδ T cells were quantified in the liver (n=5). (c) WT mice were fed with an NCD or SSD diet. At indicated time points, liver γδ T cells were restimulated *in vitro* with PMA/Ionomycin and cytokine production was measured by flow cytometry (n = 10). (d-f) WT mice were fed with a NCD or SSD diet for 2 weeks. (d) Liver γδ T cells were restimulated *in vitro* with PMA/Ionomycin and cytokine production was measured by flow cytometry (n = 5). (e) Shown is a t-SNE analysis on flow cytometry measurements of hepatic γδ T cells with the indicated markers superimposed. (f) Vγ expression was measured within γδ T cells by flow cytometry (n = 5). (g-k) WT mice were fed with an NCD or MCD diet for 2 weeks (g-i). (g) Percentage of γδ T cells was quantified in the liver by flow cytometry (n=5). (h) IL-17A production by γδ T cells was analyzed by flow cytometry (n=5). (i) Quantification of IL-17A production in indicated liver populations (n=5). (j, k) WT and *Tcrd*$^{-/-}$ mice were fed with an NCD or MCD diet for 12 weeks (j) Average weight loss of MCD-fed mice in comparison to NCD-fed controls was determined (n=4-5). (k) Fibrosis relative to steatosis was determined on Sirius-red stained histological slides. (l) WT mice were fed an SSD for 2 weeks. Next, animals were injected with biotinylated antibodies against CD45 and sacrificed after 5 minutes. Labeled cells were visualized using APC-eFluor780-conjugated streptavidin, measured by flow cytometry. Liver-resident cells were defined as eFluor-780^{-}. The percentage of liver-resident αβ, and γδ T cells is shown (n=5). (m) WT (CD45.2) mice were NCD- or SSD-fed for 2 weeks and were adoptively transferred with leukocytes from NCD (CD45.1) and SSD (CD45.1/2) co-fed animals, mixed in a 1:1 ratio. After 24h, the percentage of donor αβ and γδ T cells was determined in spleen and liver by flow cytometry (*n* = 4 – 5). (n-o) WT mice were fed an NCD or SSD for 2 weeks and for the last 4 days given BrdU in drinking water. Tissue-resident cells were defined as described under l. (n) Data show the relative increase of Ki-67^{+} γδ T cells in SSD- over NCD-fed mice (n=5). (o) Representative plots of data shown in 6f and S6n. (p) WT and *Klrk1*$^{-/-}$ mice were fed for 2 weeks with an NCD or SSD. Hepatic γδ T cells were quantified by flow cytometry (n=5). The data are representative of at least two independent experiments. Shown are means +/- s.e.m. Statistical significance was determined by ANOVA with Bonferroni post-testing (b), or unpaired t-test. Statistical significance was defined as *p<0.05; **p<0.01; ***p<0.001.

Supplementary Figure 7

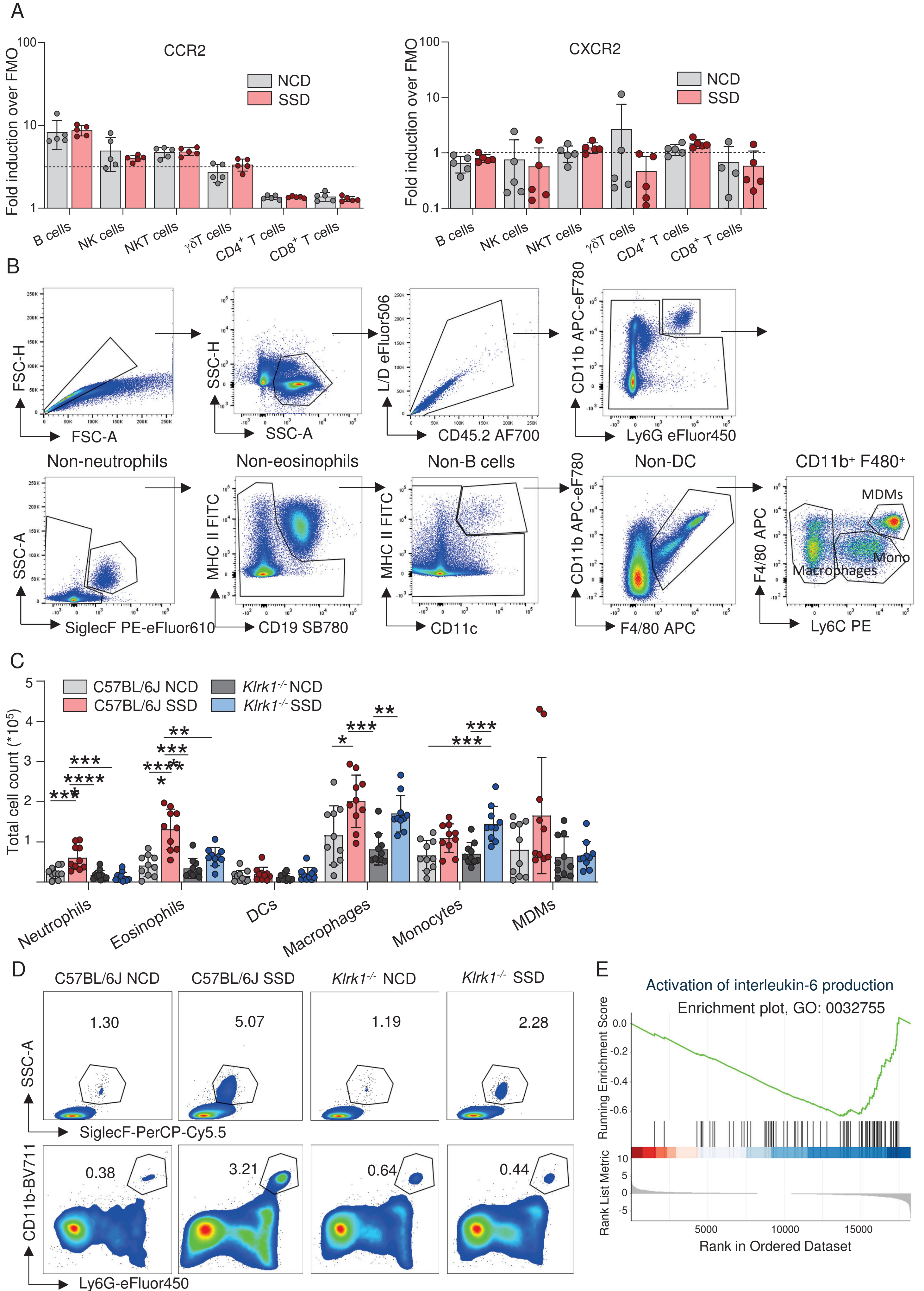

Figure S7. NKG2D mediates myeloid cell recruitment in context of NASH. (a-d) WT mice were fed with NCD or SSD for 4 weeks. (a) Expression levels of CCR2 (left), or CXCR2 (right) were determined on indicated lymphocyte populations by flow cytometry. (b) gating strategy of data shown in 7d. (c) Quantification of absolute numbers of the indicated myeloid populations (n=10). (d) Representative plots of data shown in 7d. (e) WT and *Klrk1*$^{-/-}$ mice were fed an SSD for 3 weeks and the total transcriptome of liver tissue was analyzed by RNA sequencing. Differential gene expression for genes associated with activation of IL-6 production are shown. The data are representative of one experiment using 3-5 biological replicates (e) or aleast two independent experiments (a-d). Shown are means +/- s.e.m. Statistical significance was determined by ANOVA with Bonferroni post-testing (c). Statistical significance was defined as *p<0.05; **p<0.01; ***p<0.001.

Supplementary Figure 8

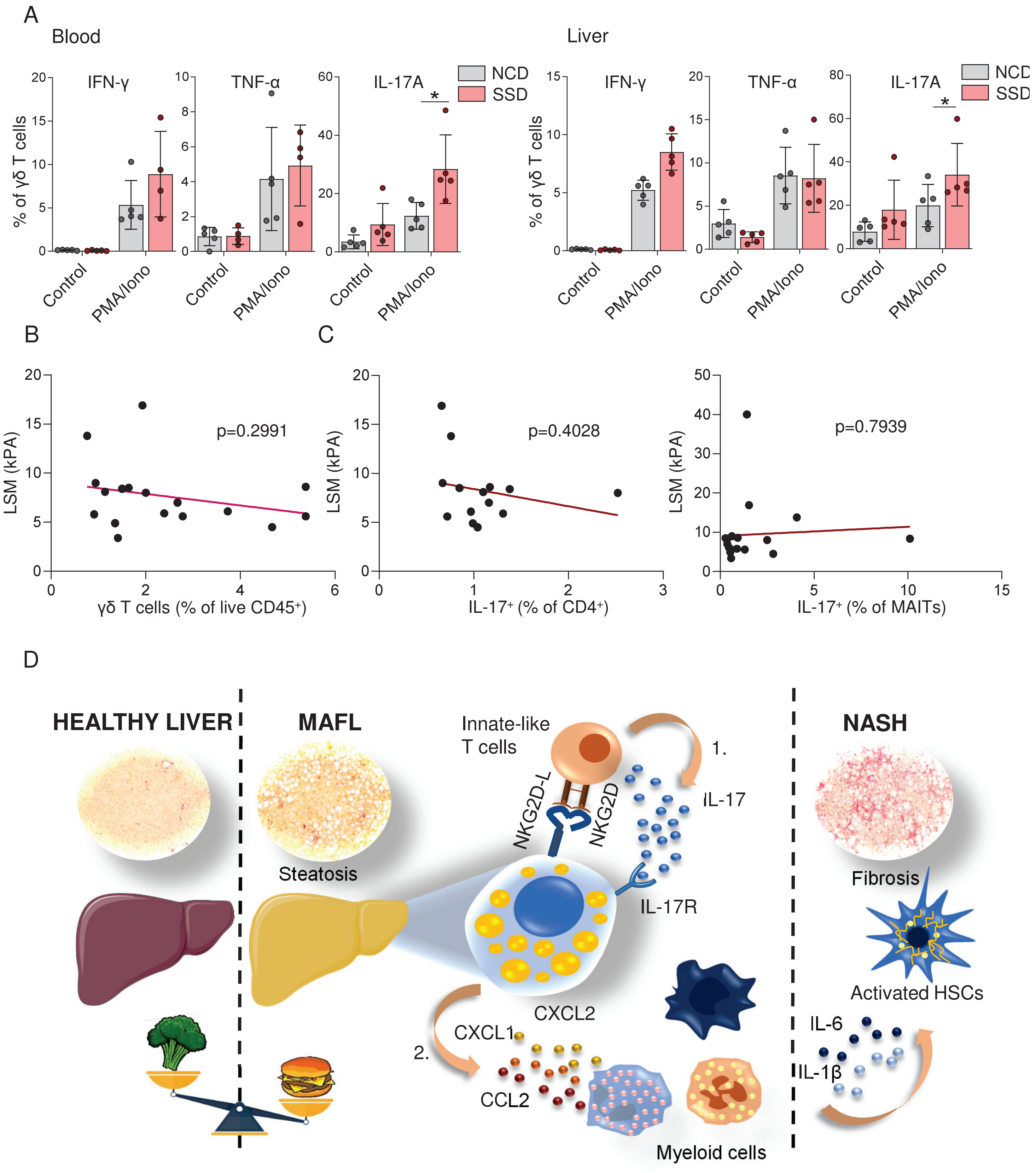

Figure S8. IL-17A and RORγt expression by γδ T cells correlates between liver and blood. (a) WT mice were fed with a NCD or SSD diet and after 2 weeks, liver and blood leukocytes were analyzed by flow cytometry. (b-c) people with MAFLD were analyzed for liver stiffness (LSM) by ultrasound measurement. Next, their peripheral blood mononuclear cells were restimulated *in vitro* with PMA/Ionomycin and cells were analyzed by flow cytometry. (b) correlation between liver stiffness measurement (LSM; an indicator of fibrosis) and the percentage of γδ T cells. (c) correlation between liver stiffness measurement (LSM; an indicator of fibrosis) and the percentage of (left) $CD4^+$ T cells and (right) MAIT cells producing IL-17A (n=18). (d) Graphical representation of the model of NASH initiation proposed by the authors. The data are representative of at least two independent experiments. (a) Shown are means +/- s.e.m. Statistical significance was determined by unpaired t-test (a) or linear regression (b-c). Statistical significance was defined as $^*p<0.05$; $^{**}p<0.01$; $^{***}p<0.001$.
